# Dysregulation of Type I Interferon (IFN-I) Signaling: A Potential Contributor to Racial Disparity in Hepatocellular Carcinoma (HCC)

**DOI:** 10.3390/cancers15174283

**Published:** 2023-08-27

**Authors:** Saranya Chidambaranathan Reghupaty, Sadia Kanwal, Rachel G. Mendoza, Eva Davis, Haiwen Li, Zhao Lai, Mikhail G. Dozmorov, Milton Omar Faison, Rafat Ali Siddiqui, Devanand Sarkar

**Affiliations:** 1 C. Kenneth and Dianne Wright Center for Clinical and Translational Research, Virginia Commonwealth University, Richmond, VA 23298, USA; chidambaranas@vcu.edu; 2 Food and Nutrition Science Laboratory, College of Agriculture, Virginia State University, Petersburg, VA 23806, USA; skanwal@vsu.edu (S.K.); hali@vsu.edu (H.L.); 3 Department of Human and Molecular Genetics, Virginia Commonwealth University, Richmond, VA 23298, USA; rachel.mendoza@vcuhealth.org; 4 Department of Microbiology and Immunology, Virginia Commonwealth University, Richmond, VA 23298, USA; davisep@vcu.edu; 5 Greehey Children’s Cancer Research Institute, University of Texas Health Science Center San Antonio, San Antonio, TX 78229, USA; laiz@uthscsa.edu; 6 Department of Biostatistics and Pathology, Virginia Commonwealth University, Richmond, VA 23298, USA; mikhail.dozmorov@vcuhealth.org; 7 Department of Biology, Virginia State University, Petersburg, VA 23806, USA; mfaison@vsu.edu; 8 Department of Human and Molecular Genetics, Massey Cancer Center, VCU Institute of Molecular Medicine (VIMM), Virginia Commonwealth University, Richmond, VA 23298, USA

**Keywords:** hepatocellular carcinoma (HCC), interferon, racial disparity, interferon-stimulated genes (ISGs), inflammation, ginger

## Abstract

**Simple Summary:**

Hepatocellular carcinoma (HCC) is the most common primary liver cancer arising from the liver cells, hepatocytes. Chronic liver inflammation plays a key role in the development of HCC. HCC is a highly fatal disease where race/ethnicity plays a vital role in determining incidence, mortality and survival rates. There is a knowledge gap in our understanding of the molecular mechanism underlying the HCC racial disparity between African-American (AA)/Black and White patients. Global gene expression analysis between AA/Black and White HCC patients identified the activation of a key inflammatory pathway in AA/Black tumors. Ginger extract (GE) is known for its anti-inflammatory properties. GE inhibited proliferation of HCC cells, and our data suggest that HCC cell lines from AA/Black patients responded better to GE compared to those from White and Asian patients. These findings suggest that AA/Black HCC patients might benefit from holistic dietary approach which includes ginger.

**Abstract:**

African-American (AA)/Black hepatocellular carcinoma (HCC) patients have increased incidence and decreased survival rates compared to non-Hispanic (White) patients, the underlying molecular mechanism of which is not clear. Analysis of existing RNA-sequencing (RNA-seq) data in The Cancer Genome Atlas (TCGA) and in-house RNA-sequencing of 14 White and 18 AA/Black HCC patients revealed statistically significant activation of type I interferon (IFN-I) signaling pathway in AA/Black patients. A four-gene signature of IFN-stimulated genes (ISGs) showed increased expression in AA/Black HCC tumors versus White. HCC is a disease of chronic inflammation, and IFN-Is function as pro-inflammatory cytokines. We tested efficacy of ginger extract (GE), a dietary compound known for anti-inflammatory properties, on HCC cell lines derived from White (HepG2), AA/Black (Hep3B and O/20) and Asian (HuH-7) patients. GE exhibited a significantly lower IC_50_ on Hep3B and O/20 cells than on HepG2 and HuH-7 cells. The GE treatment inhibited the activation of downstream mediators of IFN-I signaling pathways and expression of ISGs in all four HCC cells. Our data suggest that ginger can potentially attenuate IFN-I-mediated signaling pathways in HCC, and cells from AA/Black HCC patients may be more sensitive to ginger. AA/Black HCC patients might benefit from a holistic diet containing ginger.

## 1. Introduction

Hepatocellular carcinoma (HCC) is the most common primary liver cancer. Most HCC cases are diagnosed at advanced stages where the prognosis is dismal owing to the inherent resistance of HCC cells to conventional chemo- and radiotherapy and limited surgical and nonsurgical interventions due to underlying liver disease (such as cirrhosis) [1,2]. The most effective US Food and Drug Administration (FDA)-approved treatment for advanced nonresectable HCC is a combination of anti-PD-L1 (atezolizumab) and anti-VEGF (bevacizumab) antibodies, providing an overall response rate of 27% [3,4]. This bleak scenario mandates an analysis of underlying molecular mechanisms of HCC so that novel, targeted treatment approaches can be developed and evaluated.

For HCC, race/ethnicity plays an important role in determining incidence, mortality and survival rates. There is a statistically significant difference in incidence, mortality and 5-year survival rates between non-Hispanic Whites (White) and African Americans (AA)/Blacks for liver and intrahepatic bile duct cancer patients (Table 1) [5].

In a recent study of 1117 patients (35.9% White, 34.3% AA/Black, 29.7% Hispanic), AA/Black patients were 25% less likely to be diagnosed with early stage HCC and had a median shorter survival (10.6 vs. 16.3 months) compared to White patients [6]. AA/Black HCC patients had a significantly higher mortality than White patients (hazard ratio, 1.12; 95% CI, 1.10–1.14) even after adjustment for Barcelona Clinic Liver Cancer (BCLC) stage, Child–Pugh class, type of medical insurance and receival of treatment for HCC. Most reports have focused on socioeconomic status and a lack of access to timely care as the root cause for the discrepancy in outcomes between AA/Blacks and Whites [7,8,9,10]. However, in many cases these results are conflicting even when analyzing similar databases. Recent data suggest that race/ethnicity determines response to treatment for HCC [11], and genetic differences altered response to interferon in patients with chronic hepatitis C virus (HCV) [12]. Studies examining metabolite profiles in HCC patients support the hypothesis that underlying genetic differences contribute to outcomes from HCC [13]. Thus, underlying molecular mechanisms contribute to poorer outcomes in AA/Black patients and may be targeted to develop effective treatment.

Due to the severe toxicity of the currently available cancer treatments, there is also a demand for the development of alternative therapies with high efficacy and low side effects to treat liver cancer. Recent data indicate that over 60% of Gen Xers/Millennials are taking a more holistic approach to their diet to prevent chronic diseases, including cancer [14]. Ginger (Zingiber officinale Roscoe, *Zingiberacae*) is one of the most commonly used herbal spices that has shown several medicinal properties. The biologically active phenolic compounds in ginger, including gingerol, paradol and shogaol, have been demonstrated to possess antioxidant, anticancer, anti-inflammatory and hepatoprotective effects [15,16,17,18]. In our previous studies, we have demonstrated that ginger extract exerts potent antioxidant effects and inhibits lipid accumulation and triglyceride content in liver cancer cells [19].

In the present study, we compared gene expression profiles of AA/Black and White HCC patients and identified a significant activation of type I interferon (IFN-I) signaling pathways in AA/Black patients. IFN-Is function as pro-inflammatory and immunosuppressive cytokines. We hypothesized that because of its anti-inflammatory properties, ginger might be more effective in AA/Black HCC patients. We investigated the effects of ginger extract on proliferation and IFN-I signaling pathways in HCC cells derived from White (HepG2), Black (Hep3B and O/20) and Asian (HuH-7) patients. Our finding might pave the way for inclusion of ginger extract in the therapeutic strategy of AA/Black HCC patients.

## 2. Materials and Methods

### 2.1. Cell Lines and Chemicals

HepG2 and Hep3B cells were obtained from American Type Culture Collection, HuH-7 cells were obtained from Japanese Collection of Research Bioresources Cell Bank, and O/20 cells were developed in-house and cultured as described [20]. Dimethyl sulfoxide, methanol (HPLC grade), isopropanol, N,N,N0,N0- Tetramethyl ethylenediamine, glycine, sodium lauryl sulfate, ammonium persulfate and MTT were purchased from Sigma-Aldrich (St. Louis, MO, USA). Tris-HCl and acrylamide/bis-acrylamide (30%/0.8% *w*/*v*) were obtained from BioRad, Hercules, CA, USA, and 1-Step™ Ultra TMB-Blotting Solution was from ThermoFisher scientific, Waltham, MA, USA.

### 2.2. Analysis of TCGA RNA-sequencing (RNA-seq) Data

Level-3 gene expression data for 423 liver hepatocellular carcinoma (LIHC) samples summarized as RSEM values were obtained from Broad Firehose using the TCGA2STAT R package v. 1.2, along with the 18 clinical annotations, available for 377 samples. These clinical annotations were used to subset the samples by race. Differentially expressed genes were identified using the limma v. 3.32.6 R package [21,22]. P-values were corrected for multiple testing using the False Discovery Rate (FDR) method [23].

### 2.3. RNA Sequencing (RNA-seq) of Tumor Samples

Human HCC tumor samples were archived by Tissue and Data Acquisition and Analysis Core (TDAAC) of Massey Cancer Center, Virginia Commonwealth University. Patient information is provided in Table 2.

**Figure 1:**
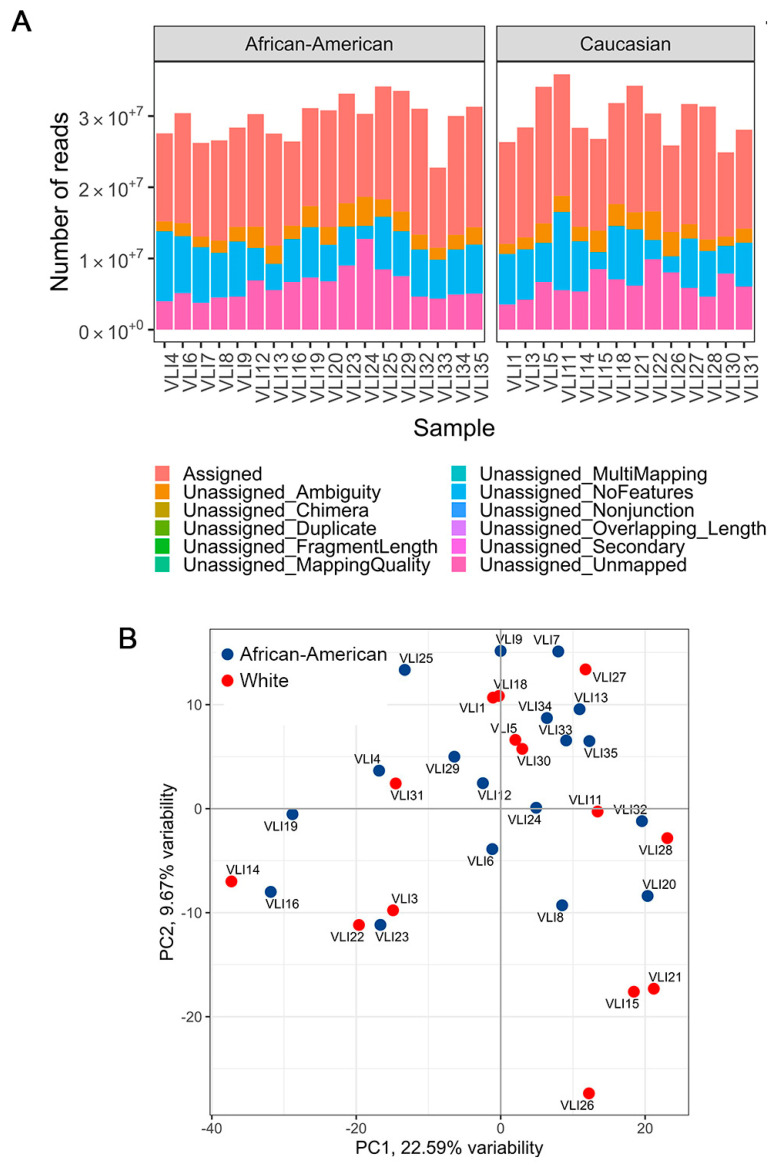
Quality assessment of RNA-seq samples. (**A**) Quality metrics of RNA-seq samples show approximately similar sequencing depth and the proportion of aligned reads. (**B**) Principal component analysis of samples, colored by race.

Total RNA from tumor tissues was isolated with MagMax-96 for Microarrays Total RNA Isolation Kit (ThermoFisher) according to the manufacturer’s recommendations. RNA integrity (RIN) and yield were assessed on an Agilent 2100 Bioanalyzer, and samples with RIN ≥ 7 were selected. Prior to the RNA-seq, ribosomal RNA was depleted from the samples using the RiboMinus™ Human/Mouse Transcriptome Isolation Kit (Thermo Fisher Scientific, Waltham, MA, USA). RNA-seq library was prepared using Illumina TruSeq stranded mRNA sample preparation kit and all samples were sequenced on the Illumina Hi-Seq 2500 according to Illumina’s sequencing-by-synthesis protocol. One sample was sequenced across three different flow cells to estimate the technical variability of sequencing runs. We obtained ~30 million 50 bp single-end reads per sample. Quality control at each processing step was performed using the FastQC tool (quality base calls, CG content distribution, duplicate levels, complexity level) [24]. Sequencing adapters were removed using Trimmomatic [25]. Reads were aligned to the latest assembly of the human genome (GRCh38/hg38) using the subread v.1.6.2 aligner [26]. Post-processing quality assessment of RNA-seq samples showed an overall good quality of genomic alignment in samples from both AA/Black and White groups (Figure 1A). Gene-level count summaries for each sample were obtained using the featureCounts v.1.6.2 software [27]. Principal Component Analysis showed a relatively uniform mixture of races across samples projected on the first two principal components (Figure 1B). RNA-seq counts were quantile-normalized by library size [28], and genes differentially expressed between AA/Black and White groups were detected using the edgeR v.3.18.1 R package [29]. We did not control age and gender to keep the analysis compatible with TCGA that did not provide age and had insufficient samples for controlling for gender. *p*-values for differentially expressed genes were corrected using a False Discovery Rate (FDR) multiple testing correction method [23]. GEO Series accession number of this dataset is GSE223795 (reviewer’s access token afwlsokcbdinlkl).

### 2.4. Total RNA Extraction, cDNA Preparation and Quantitative Real Time PCR of Tumor Samples

Total RNA was extracted from HCC tumor tissues using the QIAGEN miRNAeasy Mini Kit (QIAGEN, Valencia, CA, USA). The cDNA preparation was performed using an ABI cDNA synthesis kit (Applied Biosystems, Foster City, CA, USA). Real-time polymerase chain reaction (RT-PCR) was performed using an ABI ViiA7 fast real-time PCR system and Taqman gene expression assays according to the manufacturer’s protocol (Applied Biosystems, Foster City, CA, USA).

### 2.5. RNA in situ Hybridization (RNAscope) Assay

Fluorescent RNAscope was performed on FFPE sections with the Leica Biosystems’ BOND RX instrument following Advanced Cell Diagnostic (ACD) protocol using RNAscope LS reagent kit_Red (ACD). A 20ZZ probe targeting 2-824 of NM_002038.3 was purchased from ACD to specifically detect human IFI6 mRNA by Fast Red chromogen staining. The slides were imaged using the Phenoimager HT automated imaging system (Akoya Biosciences, Marlborough, MA, USA). H-scoring (% positive cells × Intensity) was used to quantify individual markers within each region of interest.

### 2.6. Immunohistochemistry

IHC was performed on formalin-fixed paraffin-embedded (FFPE) sections as described [30] using the following primary antibodies: OAS1 (HPA003657, Sigma, rabbit, 1:100) and MX1 (HPA0309170, Sigma, rabbit, 1:100). Images were taken using Olympus BX41 microscope. H-scoring (% positive cells × Intensity) was used to quantify individual markers within each region of interest.

### 2.7. Ethanolic Extraction of Ginger

The Ginger sample was obtained from Randolph Farm, Virginia State University (VSU). Ginger samples were cleaned with distilled water and diced into small pieces and then freeze-dried under low pressure (200 mTorr) in a freeze drier (SP Scientific, Gardiner, NY, USA). Dried samples were milled using a Scienceware Bel ArtMicro-Mill (Pequannock, NJ, USA) and then passed through a 400 μm mesh. The powder was extracted with 80% ethanol (50 mg/mL) for 18 h at ambient temperature with 250 rpm on a Scilogex SK-O330-PRO orbital shaker (Scilogex, CT, USA) followed by centrifugation at 2500× *g* 20 °C for 40 min. The ethanolic extract was evaporated in a nitrogen evaporator (Organomation Associates, Inc., Berlin, MA, USA) followed by freeze-drying overnight to remove the traces of ethanol/water residues. The dried residues were dissolved in DMSO. All extracts were kept under nitrogen and stored at −20 °C until further analysis.

### 2.8. Measurement of Cell Proliferation by MTT Assay

Cells were seeded at a density of 1 × 10^4^ cells/well in 96-well plates and incubated for 24 h. Cells were treated with GE at different concentrations and incubated for another 24 h. The next day, GE containing medium was replaced with 0.5 mg/mL MTT-culture medium [3-(4,5-dimethylthiazol-2-yl)-2,5-diphenyltetrazolium (bromide)] (Sigma, St. Louis, MO, USA) for 4 h. Then, MTT-containing media were removed and DMSO (100 µL/well) was used to dissolve blue formazan crystals. The viable cells converted MTT to formazan, which generated a blue-purple color after dissolving in 100 μL of DMSO. The developed color was measured at 570 nm using a 96-wells plate reader (Molecular Devices, San Jose, CA, USA).

### 2.9. Western Blot Analysis

Cells were seeded (2 × 10^5^) in a six well plate for 24 h and were treated with GE at 150 and 200 (µg/mL) for 24 h followed by washing with pre-cooled PBS. Ice cold RIPA buffer with protease/phosphatase was added for the cell lysis. The lysates were collected and centrifuged at 13,000× *g* for 30 min at 4 °C. The total protein concentration in cell lysates was measured by the Bradford method. The cell extracts were separated by SDS polyacrylamide gels (8–10%), and then, the protein was transferred to a polyvinylidene difluoride membrane (Millipore, Bedford, MA, USA). The membrane was blocked with 5% skim milk, incubated with indicated primary and appropriate secondary antibodies (anti-rabbit IgG-HRP conjugates), and protein bands were detected using 1-Step Ultra TMB-Blotting Solution (Thermo Scientific). STAT1 (D1K9Y) Rabbit mAb, Phospho-STAT1 (Tyr701) (58D6) Rabbit mAb, STAT2 (D9J7L) Rabbit mAb, Phospho-STAT2 (Tyr690) (D3P2P) Rabbit mAb, JAK1 (6G4) Rabbit mAb, Phospho-JAK1(Tyr1034/1035) (D7N4Z) Rabbit mAb, TYK2 (D4I5T) Rabbit mAb, Phospho-TYK2 (Tyr1054/1055) (D7T8A) Rabbit mAb, β-Actin (13E5) Rabbit mAb and Anti-rabbit HRP-linked antibodies were purchased from Cell Signaling Technology (Danvers, MA, USA). Densitometric analysis was performed by Image J software version 1.44.

### 2.10. Measurement of ISG Expression by Real-Time Polymerase Chain Reaction (RT-PCR)

Cells were seeded in a 6-well plate at a density of 2 × 10^5^ for 24 h. The next day, cells were treated with GE (150 and 200 µg/mL) or vehicle control (DMSO) for 24 h. The expression of four ISGs (MX1, OAS1, IFI6 and ISG15) was determined using a quantitative RT-PCR. Briefly, total RNA was extracted from all the above-mentioned treated cells using the RNeasy mini kit (Qiagen, Germantown, MD, USA). The concentration of the RNA was determined by NanoDrop 2000 (ThermoFisher, Washington, DC, USA). First-strand cDNA was synthesized from total RNA using the RT2 Easy first strand kit (Qiagen, Germantown, MD, USA). ISG mRNA expression levels were analyzed via real-time PCR (QuantStudio 3, Applied Biosystems instrument, Waltham, MA, USA) under the following reaction conditions: initial denaturation at 95 °C for 5 m, followed by 40 cycles of denaturation at 95 °C for 30 s, annealing at 60 °C for 30 s, and lastly elongation at 72 °C for 30 s. The relative gene expression was quantified using the 2−ΔΔCt method. All the targeted gene levels were normalized against GAPDH. The sequence of the forward (F) and reverse (R) primers are MX1-F: 5′-AGGACCATCGGAATCTTGAC-3′, MX1-R: 5′-TCAGGTGGAACACGAGGTTC-3′; OAS1-F: 5′-AGATCAATGAGCCCTGCATAAACC-3′, OAS1-R: 5′-ATTGACAGTGCTGTTAACATCATC-3′; IFI6-F: 5′-TGATGAGCTGGTCTGCGATCCT-3′, IFI6-R: 5′-GTAGCCCATCAGGGCACCAATA-3′; ISG15-F: 5′-GGACAAATGCGACGAACCTCT-3′, ISG15-R: 5′-GCCCGCTCACTTGCTGCTT-3′; GAPDH-F: 5′-TGAGTACGTCGTGGAGTCCA-3′, GAPDH-R: 5′-TAGACTCCACGACATACTCA-3′.

### 2.11. Statistical Analysis

All values are expressed as mean ± standard deviation. A comparison of the results was performed using one-way ANOVA and Tukey’s multiple comparison tests (GraphPad Prism software 9.5.1, La Jolla, CA, USA). Statistically significant differences between groups were defined as *p*-values less than 0.05.

## 3. Results

### 3.1. Analysis of TCGA Database

**Figure 2:**
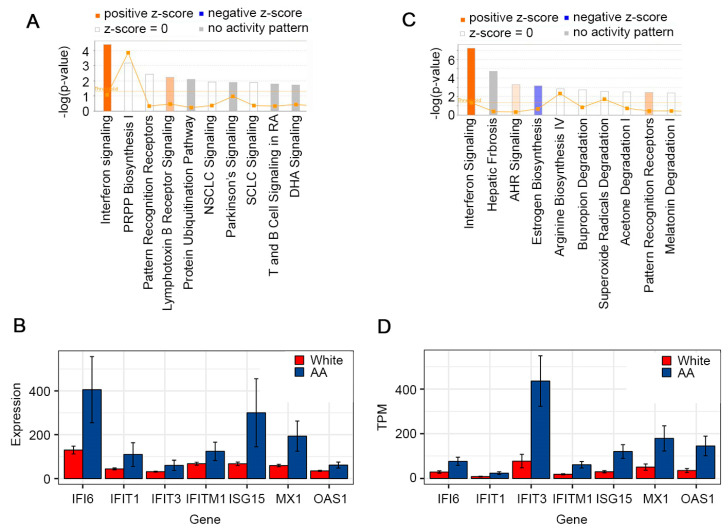
IFN-I signaling is activated in AA/Black HCC patients compared to White HCC patients. (**A**) Canonical pathways modulated in AA/Black HCC patients in TCGA database. PRPP: phosphoribosyl pyrophosphate; NSCLC: non-small cell lung cancer; SCLC: small cell lung cancer; DHA: docosahexaenoic acid. (**B**) Bar plots of expression of selected genes in White and AA/Black HCC tumor samples in TCGA. Standard error bars are shown. (**C**) Canonical pathways modulated in AA/Black HCC patients in VCU samples. AHR: arylhydrocarbon receptor. (**D**) Bar plots of expression of selected genes in White and AA/Black HCC tumor samples obtained in VCU. Standard error bars are shown. TPM: transcripts per million.

To understand the potential molecular mechanisms responsible for decreased survival in AA/Black patients with HCC, gene expression profiles between HCC samples from White and AA/Black patients in TCGA database were compared. Only 17 samples from AA/Black patients were obtained compared to 184 samples from White patients, indicating that the TCGA database is a biased database for performing disparity-related studies. Given the small number of AA/Black samples, we were underpowered in the detection of differentially expressed genes. Therefore, we chose to utilize the unadjusted *p*-value cutoff of 0.01, at the expense of potential noise that may diminish the significance of functional enrichment analysis. We identified 278 differentially expressed genes, 205 of them were upregulated and 73 were downregulated in AA/Black patients (Table S1). These differentially expressed genes (DEGs) were subjected to ingenuity pathway analysis (IPA) to identify which canonical pathways are differentially modulated in AA/Black patients. A z-score of >2 indicates pathway activation. The IFN-I signaling pathway was identified to be the most significantly enriched pathway in AA/Black HCC patients (Figure 2A). In this dataset, the examination of expression levels of ISGs confirmed their upregulation in AA/Black patients compared to White patients (Figure 2B).

### 3.2. In-House RNA-seq and Analysis of White vs. AA/Black HCC Samples

The RNA-seq analysis of archived HCC tumors from 14 White and 18 AA/Black patients detected 283 DEGs, with 186 being downregulated and 97 were upregulated in AA/Black vs. White patients (Table S2). Since our preliminary results were run on a relatively small cohort of highly variable human gene expression data, thus being underpowered, we used the unadjusted *p*-value cutoff of 0.01. The IPA analysis of DEGs identified that the only canonical pathway significantly activated in AA/Black HCC patients is the IFN-I signaling pathway, correlating with the TCGA analysis (Figure 2C). Similar to the TCGA analysis, the ISGs were upregulated in these cohorts as well (Figure 2D).

### 3.3. Validation in Patient Samples

**Figure 3:**
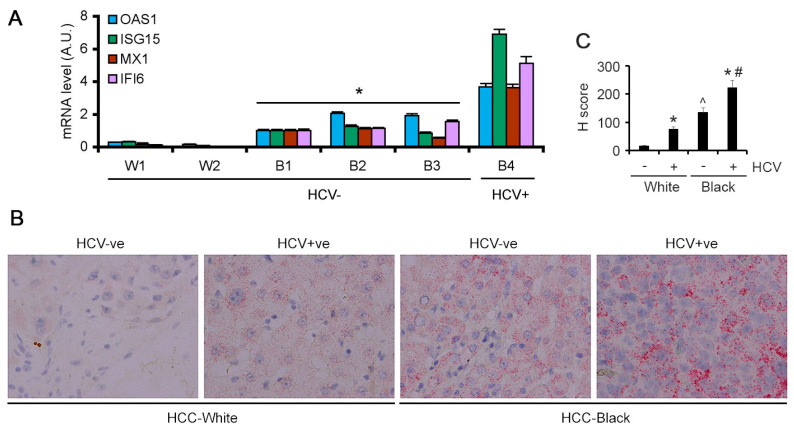
ISGs are overexpressed in AA/Black HCC patients. (**A**) Taqman qRT-PCR of the indicated genes in tumor samples from two White patients (W1 and W2) and four AA/Black patients (B1–B4) normalized by GAPDH levels. The levels in B1 were considered as 1. Data represent mean ± SEM; * *p* < 0.01 vs. W1 or W2. (**B**) Representative images of RNA in situ hybridization for IFI6 in the indicated samples. (**C**) Quantification of RNA in situ hybridization for IFI6. Data represent mean ± SEM; * *p* < 0.01 vs. corresponding HCV-ve; ^ *p* < 0.01 vs. HCV+ve White; # *p* < 0.01 vs. HCV+ve White.

The findings obtained from archived HCC samples were validated in fresh HCC samples obtained by TDAAC. We selected four ISGs, OAS1, ISG15, MX1 and IFI6, for further validation by Taqman Q-RT-PCR in the HCC tumor tissues. These four ISGs were selected because they showed strong statistically significant increased levels in AA/Black HCC patients compared to White HCC patients in both TCGA analysis and our own RNA-seq analysis. Since these four ISGs exert strong antiviral properties and are induced by HCV infection, we focused our analysis only on HCV-negative HCC patients. In two HCV-negative White HCC tumors the levels of the four ISGs were very low (Figure 3A). In three HCV-negative AA/Black HCC tumors, their expression levels were significantly and robustly higher. In an HCV-positive AA/Black HCC tumor, their expression was further higher (Figure 3A). These findings indicate that these four ISGs might have an important role in regulating hepatocarcinogenesis in AA/Black HCC patients independent of their HCV status.

The findings from Q-RT-PCR were validated by additional methods. Because of the lack of a suitable commercially available antibody for IFI6, we performed RNAScope RNA in situ hybridization (ISH). IFI6 mRNA expression was negligible in the HCV-negative White HCC patients (Figure 3B). In the HCV-positive White HCC patients, IFI6 mRNA expression was detectable. However, in the HCV-negative AA/Black HCC patients, IFI6 mRNA levels were higher than those in HCV-positive White HCC patients, and in the HCV-positive AA/Black HCC patients, it was further augmented (Figure 3B,C).

**Figure 4:**
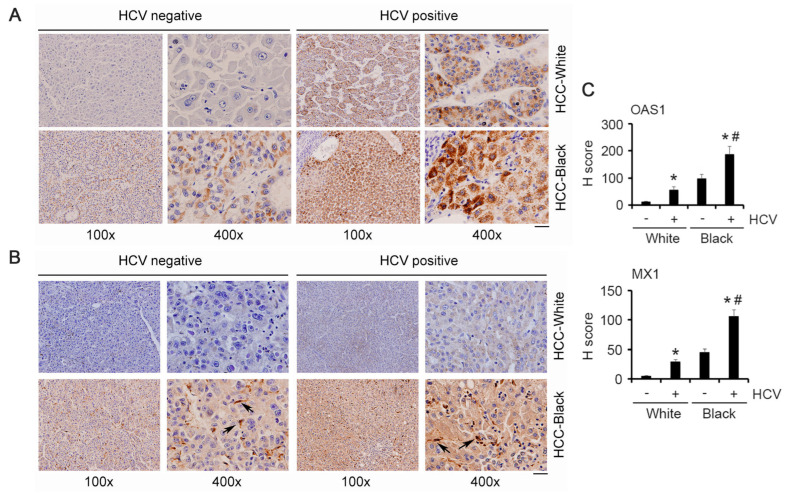
OAS1 and MX1 are overexpressed in AA/Black HCC patients. Representative images of immunohistochemistry for OAS1 (**A**) and MX1 (**B**) in the indicated samples. Arrows indicate Kupffer cells. Scale bar: 20 μm. (**C**) Quantification of IHC for OAS1 and MX1. Data represent mean ± SEM; * *p* < 0.01 vs. corresponding HCV-ve; # *p* < 0.01 vs. HCV+ve White.

We checked OAS1 and MX1 expression levels in the FFPE sections of the HCV-negative and HCV-positive White and AA/Black HCC samples by immunohistochemistry. HCC-negative White samples did not stain for OAS1 and MX1, while HCC-negative AA/Black samples showed uniform staining for OAS1 and MX1 (Figure 4A–C). HCV-positive White samples stained for OAS1 and MX1 at an intensity similar to that of the HCV-negative AA/Black samples. HCV-positive AA/Black sample showed strong and intense staining for OAS1 and MX1 (Figure 4A–C). Interestingly, in AA/Black samples, in addition to the tumor cells, MX1 staining was observed in tumor microenvironment cells, mostly Kupffer cells (arrows in Figure 4B), indicating potential involvement of MX1 in regulating Kupffer cell function, hence inflammation.

### 3.4. Effect of Ginger Extract (GE) on the Viability of HCC Cell Lines

**Figure 5:**
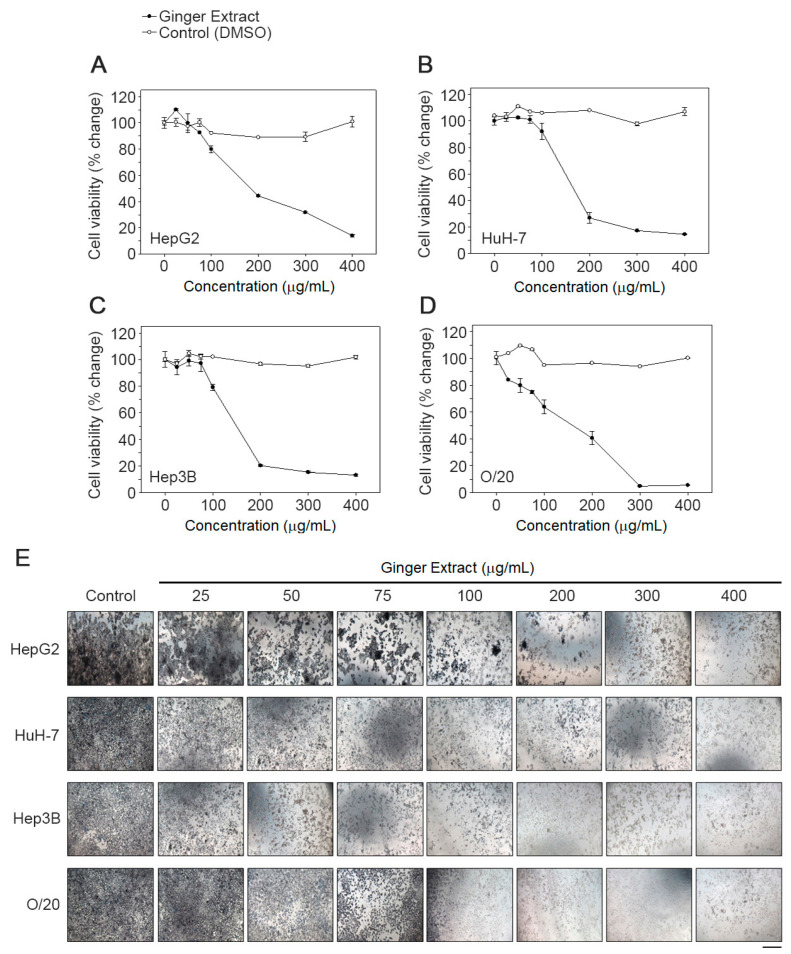
GE inhibits proliferation of HCC cells. A dose response effect of GE on (**A**) HepG2, (**B**) HuH-7, (**C**) Hep3B and (**D**) O/20 cell proliferation was determined using an MTT assay as described in the text. Each data point represents the means of 3 independent experiments with bars showing the standard deviation. The IC50 values were calculated and compared using one-way ANOVA. (**E**) Effect of GE on cell morphology. The images were taken at a magnification 100×. Images are representative of three independent experiments. Scale bar: 10 μm.

We next checked the effects of GE on HCC cells because of ginger’s anti-inflammatory properties. Treatment with GE (25, 50, 75, 100, 200, 300 and 400 µg/mL) potentiated antiproliferative effect in a dose-dependent manner. HCC cell lines derived from White (HepG2) and Asian (HuH-7) patients exhibited a similar IC50 of 177 ± 5 µg/mL and 172 ± 5 µg/mL, respectively (Figure 5A,B). AA/Black patient-derived HCC cells, Hep3B and O/20, were more sensitive to GE treatment and exhibited an IC50 of 156 ± µg/mL and 160 ± µg/mL, respectively (Figure 5C,D). The morphology of the cells was significantly deformed in GE-treated cells compared to the untreated cells (Figure 5E). Control cells were more regular and densely packed in comparison to the GE-treated cells. Particularly, at higher concentrations of GE, most of the cells shrank, and only few live cells remained attached.

### 3.5. Effect of GE Treatment on JAK/STAT Signaling Pathway

**Figure 6:**
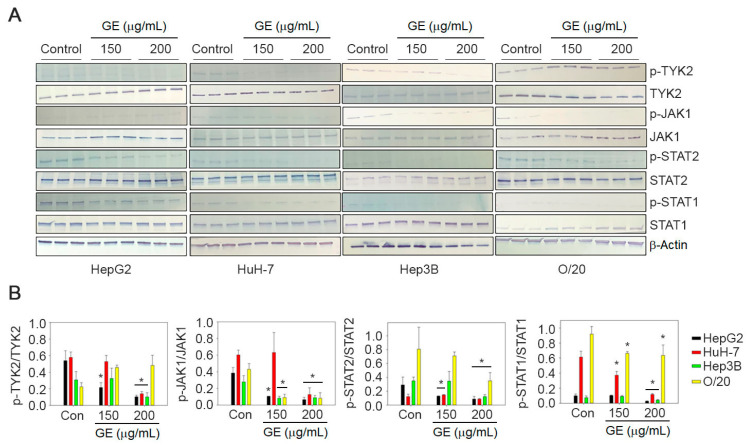
GE inhibits JAK/STAT signaling pathway in HCC cells. (**A**) HepG2, HuH-7, Hep3B and O/20 cells were treated with GE at 150 and 200 µg/mL. The cell lysates were immunoblotted with phospho- and unphospho-TYK2, JAK1, STAT2 and STAT1 antibodies. β-Actin was used as a loading control. Representative images are shown. Full pictures of the Western blots and the densitometry scans are presented in File S1. (**B**) Graphical representation of quantification of the Western blot analysis. Data are expressed as mean ± SEM; * *p* < 0.05 versus control for each gene in each cell line.

In the canonical IFN-I signaling pathway, the activation of JAK1 and TYK2 leads to the phosphorylation of STAT1 and STAT2. We evaluated the effect of GE on these downstream effector molecules. Total unphosphorylated TYK2, JAK1, STAT1 and STAT2 were expressed in all four HCC cell lines (Figure 6). The untreated cells showed detectable presence of tyrosine phosphorylated TYK2, JAK1, STAT1 and STAT2, which was variably reduced on GE treatment at 150 µg/mL and in most cases completely inhibited with GE treatment at 200 µg/mL (Figure 6).

### 3.6. Expression Level of ISGs after GE Treatment

**Figure 7:**
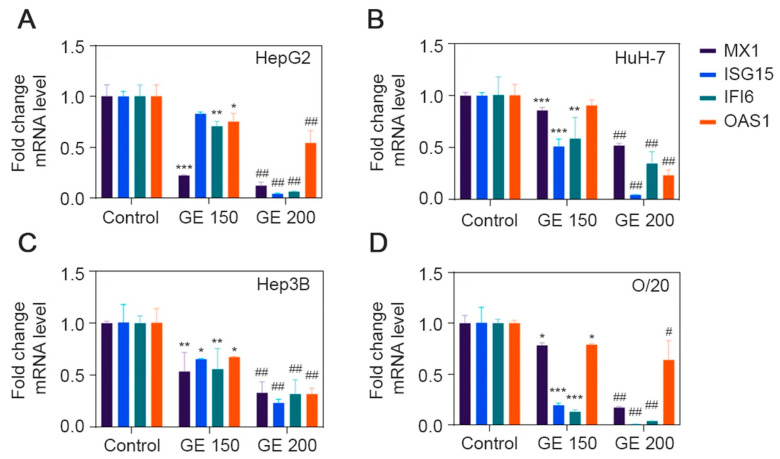
GE inhibits expression of ISGs in HCC cell lines. Expression level of MX1, ISG15, IFI6 and OAS1 after GE treatment at 150 and 200 µg/mL were determined in (**A**) HepG2, (**B**) HuH-7, (**C**) Hep3B and (**D**) O/20 cell lines. Data are expressed as mean ± SEM. Relative fold change differences were analyzed using two-way ANOVA Tukey’s multiple comparison test; * *p* < 0.05, ** *p* < 0.01, *** *p* < 0.001, Control group vs. GE (150 µg/mL); # *p* < 0.01, ## *p* < 0.001, Control group vs. GE (200 µg/mL).

We evaluated the effect of GE treatment on the mRNA expression levels of four ISGs, MX1, ISG15, IFI6 and OAS1 (Figure 7). All four ISGs could be detected in all four HCC cell lines under untreated condition, and GE treatment significantly inhibited their expression at a variable level in all four cell lines (Figure 7). MX1 was significantly inhibited, by 80–90% (*p* < 0.001), in HepG2 cells at both 150 and 200 μg/mL dose, while it was inhibited by only 50% in HuH-7 cells at 200 μg/mL. MX1 inhibition at 150 and 200 μg/mL was 50% and 70%, respectively, for Hep3B cells and 20% and 80%, respectively, for O/20 cells. In HepG2 cells, ISG15 was not affected by 150 μg/mL GE but significantly inhibited by 80–90% (*p* < 0.001) at 200 μg/mL. In HuH-7 cells, an inhibition of 40–50% (*p* < 0.001) and 90% (*p* < 0.001) was observed at 150 and 200 μg/mL of GE, respectively. ISG15 inhibition at 150 and 200 μg/mL was 30% and 70%, respectively, for Hep3B cells and 80% and 95%, respectively, for O/20 cells. IFI6 inhibition at 150 and 200 μg/mL was 20% and 90%, respectively, for HepG2 cells, 40% and 60%, respectively, for HuH-7 cells, 50% and 70% for Hep3B cells, respectively, and 85% to 95%, respectively, for O/20 cells. Among the four ISGs, OAS1 showed the least change upon GE treatment. At 150 μg/mL, HepG2, HuH-7, Hep3B and O/20 cells showed 20%, 0%, 30% and 20% inhibition in OAS1, respectively, while at 200 μg/mL, the inhibitions were 50%, 70%, 60% and 20%, respectively. Increasing GE concentration did not affect OAS1 inhibition in O/20 cells.

## 4. Discussion

There are a single IFNβ gene and 13 IFNα genes in the human IFN-I family [31]. IFN-Is bind to IFNαR1 and IFNαR2 dimeric receptors resulting in the activation of TYK2 and JAK1 which in turn phosphorylate and activate STAT1 and STAT2. IFN-stimulated gene factor 3 (ISGF3) complex is formed by STAT1, STAT2 and IFN regulatory factor 9 (IRF9). The promoters of IFN-stimulated genes (ISGs) contain IFN-stimulated response element (ISRE) sequences to which ISGF3 binds and regulates transcription. IFN-Is are induced by viral infection and various cellular stresses and function in the innate immune response providing a strong antiviral response. In the context of tumors, IFN-Is are generated both by tumor cells and tumor microenvironment cells and exert both pro- and antitumor effects [32].

Chronic inflammation, caused by hepatitis B or hepatitis C virus infection, chronic alcoholism or nonalcoholic fatty liver disease (NAFLD), is a major causative event in the pathogenesis of HCC [33]. For both chronic virus infection and cancer, IFN-Is are being identified as central drivers of chronic inflammation and immunosuppression [31]. IFN-Is activate dendritic cells (DCs) to cross-prime tumor-specific T cells and thus exhibit a beneficial effect in cancer [34,35]. IFN-Is also directly inhibit cancer cells, including HCC cells, by restricting proliferation and inducing apoptosis and senescence [32,36]. However, IFN-Is can also have a negative impact on cancer by promoting negative feedback and immunosuppression. Both in the context of virus infection and cancer, sustained IFN-I signature is associated with chronic inflammation along with increases in immune checkpoint molecules, such as PD-1, PD-L1, IL-10 and IDO1 (indoleamine 2,3 dioxygenase 1), resulting in immunosuppression [37,38,39,40,41,42,43,44]. The PD-1/PDL-1 pathway antagonizes the activation signaling pathway, thereby mediating T-cell exhaustion [45,46,47,48], and IFN-Is directly promote PD-1 transcription and diminish the duration of T-cell-mediated immunity [43]. In the case of virus infection, including HCV infection, there is initial robust IFN-I production followed by a decrease to levels observed in uninfected conditions. However, the detection of IFN-I-dependent ISG signature indicates that there is continuous production of IFN-Is [49,50]. The mechanism by which the almost undetectable IFN-Is continue to robustly impact ISG expression remains to be determined. It might be possible that IFN-Is are produced at points of cell–cell interaction. Additionally, transcriptional and/or epigenetic reprogramming might facilitate increased sensitivity to IFNR activation in the presence of small amounts of IFN-Is. In the RNA-seq analysis, we did not observe any difference in IFN-I levels between Black and White HCC patients. The observation that IFN-I signature is increased in Black HCC patients vs. White HCC patients irrespective of their HCV status generated the following hypotheses: (1) High ISG levels might be due to the unmeasurable levels of IFN-Is as seen in chronic virus infection. (2) Persistent high ISG levels might cause sustained and increased inflammation thereby leading to HCC. (3) High ISG levels might induce immunosuppression by increasing immune checkpoint molecules, such as PD-1, PD-L1, IL-10 and IDO, thus favoring HCC.

We focused on a four-gene ISG signature involving OAS1, ISG15, MX1 and IFI6, because they showed strong statistically significant increased levels in AA/Black HCC patients compared to White HCC patients. 2′,5′-Oligoadenylate synthetase (OAS1) generates 2′,5′-oligoadenylate (2–5 A) from ATP in response to stimulation by dsRNA activators [51]. The 2–5 A binds to the latent form of RNase L which then dimerizes to form a potent endoribonuclease that degrades single-stranded viral or cellular RNAs. A complete innate antiviral response requires the OAS/RNase L pathway. However, there is an extreme paucity of information on the role of OAS1 in regulating a cancer phenotype. Downregulation of OAS1 in human breast cancer cell line MDA-MB-231T resulted in decreased in vitro motility [52]. Polymorphisms in OAS1 have been shown to be associated with advanced prostate cancer although the molecular mechanism has not been elucidated [53,54]. ISG15 is a small ubiquitin-like modifier which gets bound to intracellular target proteins by sequential action of three enzymes, a process known as ISGylation which is similar to ubiquitination [55]. ISGylation of intracellular proteins protects them from degradation via the ubiquitin/26S proteasome pathway [56]. ISG15 regulates antiviral properties of type I IFNs and thus plays an important role in innate immunity [57]. However, ISG15 is overexpressed in many cancers, and in HCC, ISG15 has been implicated to promote tumorigenesis and metastasis by stabilizing the antiapoptotic protein Survivin [58]. IFI6 belongs to the FAM14 protein family and is localized in the mitochondria, where it functions to preserve mitochondrial membrane potential and thus implicated to antagonize TRAIL-, IFN- and chemotherapeutic-induced intrinsic apoptosis [59,60,61,62,63,64]. IFI6 has also been shown to mediate mutated N-Ras-induced transformation and melanoma growth, and the knockdown of IFI6 resulted in DNA replication stress due to dysregulated DNA replication via E2F2, resulting in senescence or apoptosis [64]. The regulatory role of IFI6 in HCC has not been studied yet. Mx proteins are members of the family of large GTPases, which include dynamins [65]. MX1 plays an important role in the cellular antiviral response. As yet, little is known about the regulatory role of MX1 in cancer. Thus, there is a major gap in the knowledge and understanding of the role of these four ISGs, which opens up new avenues of research.

Based on the bioinformatics data, we checked downstream key regulators of IFN-I signaling pathways in HCC lines derived from White, Asian and AA/Black patients and investigated the effect of ginger extract on these cell lines. We observed that ginger treatment remarkably reduced cell viability and increased cell death in a dose-dependent manner. Consistent to our hypothesis, our findings revealed that HCC cell lines derived from AA/Black patients (Hep3B and O/20) were more sensitive to GE treatment compared to White (HepG2) or Asian (HuH-7) HCC cell lines. Further, in all four HCC cell lines, we observed basal activation of IFN-I signaling mediators with constitutive activation of ISG expression, and GE treatment inhibited both the activation of molecules in the JAK/STAT pathway as well as ISG mRNA expression, albeit at a variable level. The variable effects of GE on different HCC lines may be due to the relative role of different genes in IFN-I signaling in cell survival and apoptosis. Furthermore, the effect of GE on IFN-I signaling in HCC lines may be time, intensity and/or context-dependent. Based on our current findings, we could not make a definitive conclusion that IFN-I inhibition was more pronounced in AA/Black HCC cell lines compared to that in the White or Asian cell line. We need to include more cell lines in our study, which is complicated by the lack of commercially available HCC cell lines from White and AA/Black backgrounds. We are expending efforts to generate such cell lines which will be valuable tools in interrogating molecular mechanisms of racial disparity in HCC and developing treatment strategies.

## 5. Conclusions

In summary, we identified the activation of IFN-I signaling in AA/Black HCC patients, using bioinformatics tools, and consistently with our hypotheses experimentally validated that these pathways may be differentially sensitive to treatment between AA/Black and White or Asian HCC patients. Considering the role of pro-inflammatory and immunosuppressive functions of IFN-Is, AA/Black HCC patients might benefit from combination of dietary anti-inflammatory agents and chemo/immunotherapy.

## Figures and Tables

**Table 1:** Incidence, mortality and 5-year survival rates for liver and intrahepatic bile duct cancer.

	Incidence Rate per 100,000, 2009 to 2013			Death Rate per 100,000, 2010 to 2014			5-Year Survival (%), 2006 to 2012		
Race/Ethnicity	All	Male	Female	All	Male	Female	All	Male	Female
White	6.3	9.7	3.3	5.5	8.0	3.3	20.1	20.2	19.8
AA/Black	10.2 *	16.8 *	5.0 *	8.4 *	13.3 *	4.6 *	16.3 *	15.5 *	18.8

* *p* < 0.05. Sources: Incidence: North American Association of Central Cancer Registries, 2016. Survival: Surveillance, Epidemiology, and End Results (SEER) Program, SEER 18 registries, 2016. Mortality: National Center for Health Statistics, Centers for Disease Control and Prevention, 2016.

**Table 2:** HCC patient information.

RNA Alias	Race	Gender	Age at Dx	HCV	HBV
VLI1	Caucasian	Male	70	No	No
VLI5	Caucasian	Male	78	No	No
VLI14	Caucasian	Male	74	No	No
VLI15	Caucasian	Male	39	No	No
VLI22	Caucasian	Male	19	No	No
VLI28	Caucasian	Male	75	No	No
VLI26	Caucasian	Female	83	No	No
VLI27	Caucasian	Female	80	No	No
VLI3	Caucasian	Male	54	Yes *	No
VLI11	Caucasian	Male	58	Yes *	No
VLI21	Caucasian	Male	68	Yes *	No
VLI30	Caucasian	Male	65	Yes	No
VLI31	Caucasian	Male	51	Yes *	No
VLI18	Caucasian	Female	61	Yes *	No
VLI8	African American	Male	73	No	No
VLI25	African American	Female	62	No	No
VLI4	African American	Male	56	Yes	No
VLI6	African American	Male	61	Yes	No
VLI13	African American	Male	60	Yes *	No
VLI16	African American	Male	64	Yes *	No
VLI20	African American	Male	52	Yes *	No
VLI23	African American	Male	65	Yes *	No
VLI24	African American	Male	64	Yes *	No
VLI29	African American	Male	57	Yes	No
VLI32	African American	Male	60	Yes	No
VLI33	African American	Male	66	Yes *	No
VLI7	African American	Female	58	Yes	No
VLI9	African American	Female	65	Yes *	No
VLI12	African American	Female	62	Yes *	No
VLI19	African American	Female	51	Yes *	No
VLI34	African American	Female	69	Yes *	No
VLI35	African American	Female	74	Yes	No

The asterisk indicates active HCV infection at the time of HCC diagnosis. All other HCV-positive patients had a history of HCV infection, but HCV was not detected at the time of HCC diagnosis.

## Data Availability

GEO Series accession number of this dataset is GSE223795 (reviewer’s access token afwlsokcbdinlkl).

## Supplementary Materials

The following supporting information can be downloaded at https://www.mdpi.com/article/10.3390/cancers15174283/s1. Table S1: Differentially expressed genes between 17 AA/Black and 184 White HCC samples from TCGA; Table S2: Differentially expressed genes between 18 AA/Black and 14 White HCC samples collected in-house. File S1: Full pictures of the Western blots and the densitometry scans.
