# Supplementary material for: Dysregulation of Type I Interferon (IFN-I) Signaling: A Potential Contributor to Racial Disparity in Hepatocellular Carcinoma (HCC)
Source: Cancers (Basel). 2023 Aug 27;15(17):4283. doi: 10.3390/cancers15174283 (PMC10486472; doi:10.3390/cancers15174283)

Western Blot Raw images

Revised

TYK2

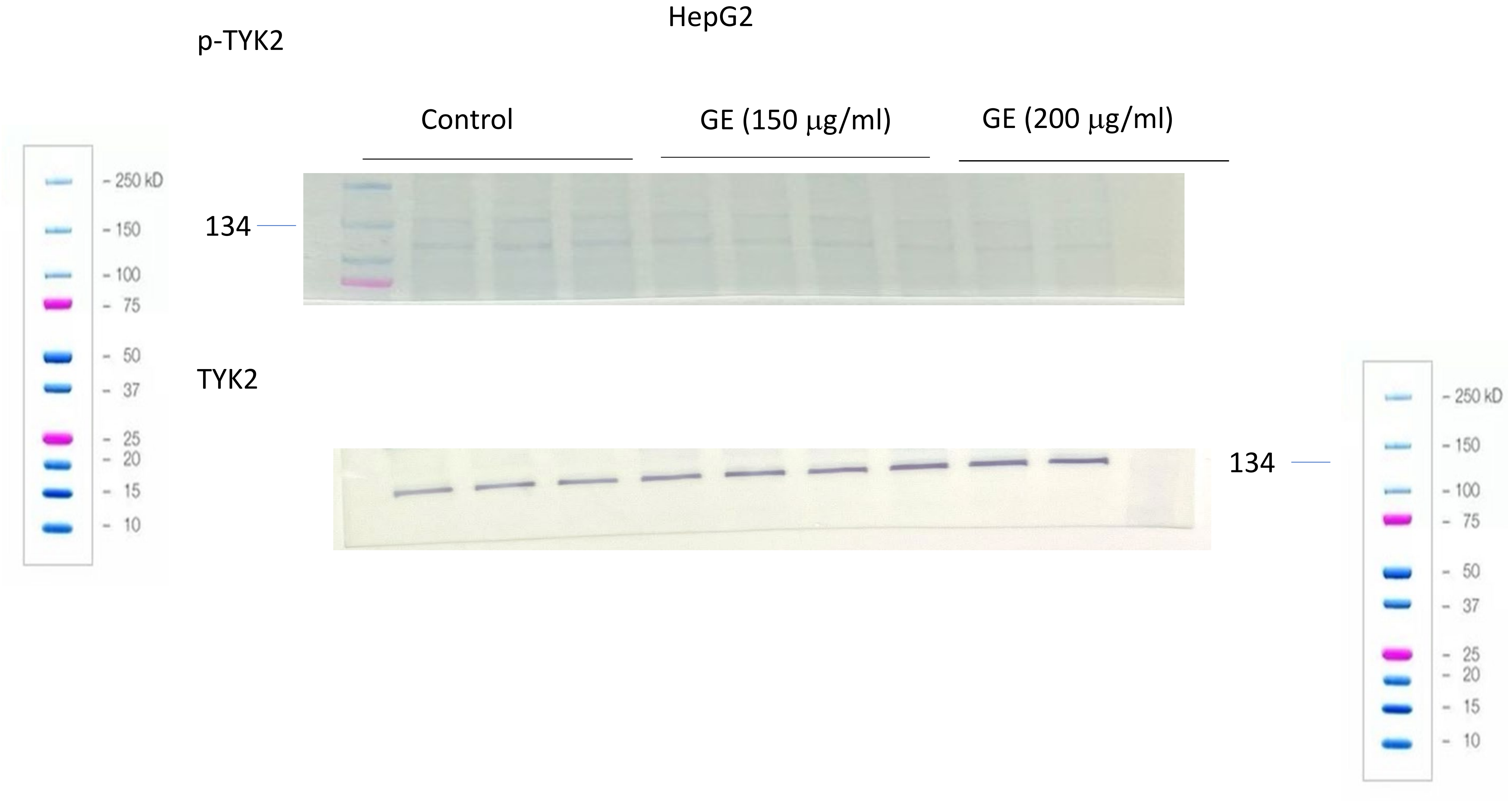
HepG2
p-TYK2
Control
GE (150 μg/ml)
GE (200 μg/ml)
134
TYK2
134
250 kD
150
100
75
50
37
25
20
15
10

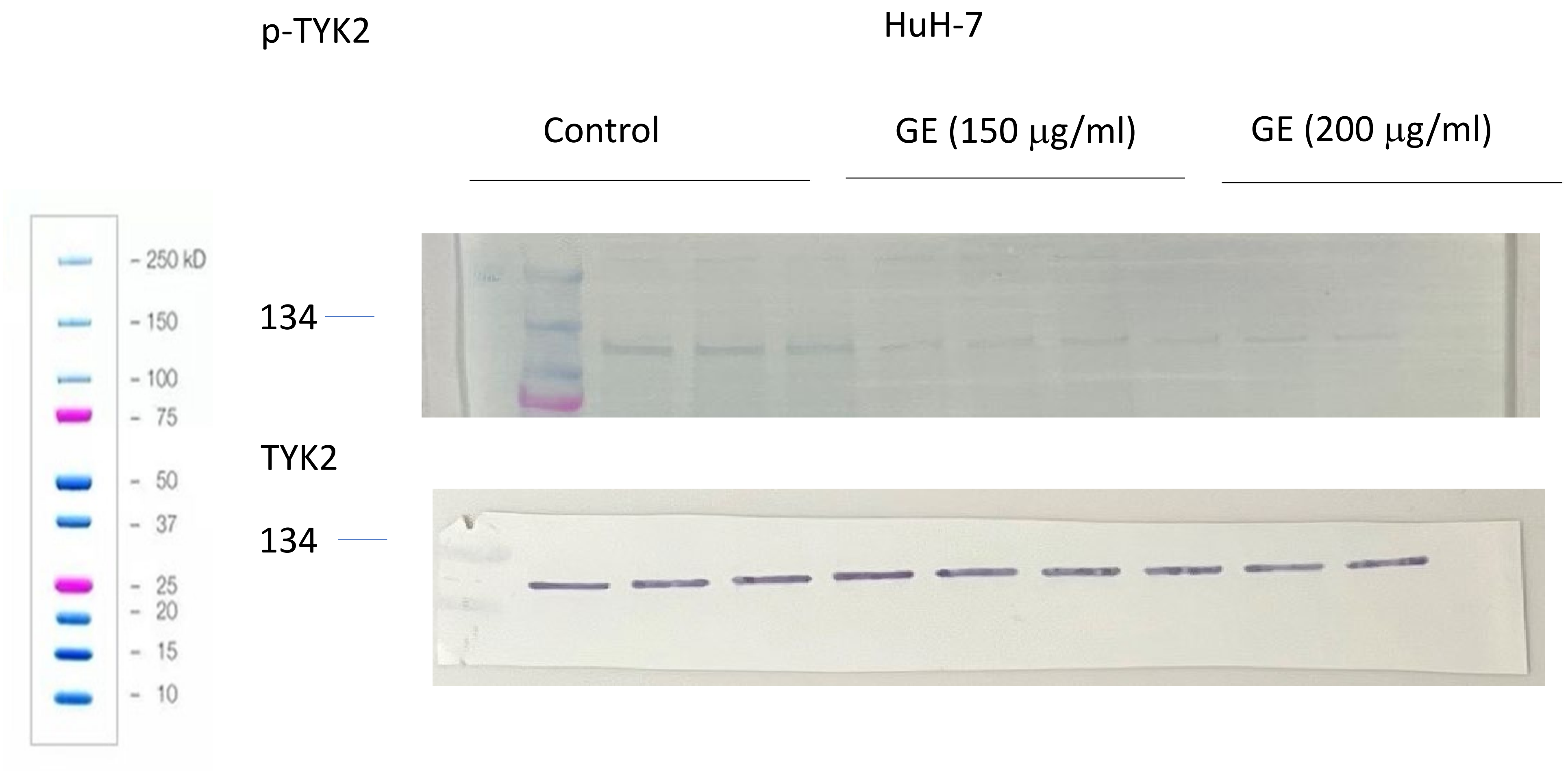
p-TYK2
HuH-7
Control
GE (150 μg/ml)
GE (200 μg/ml)
250 kD
150
100
75
50
37
25
20
15
10
134
TYK2
134

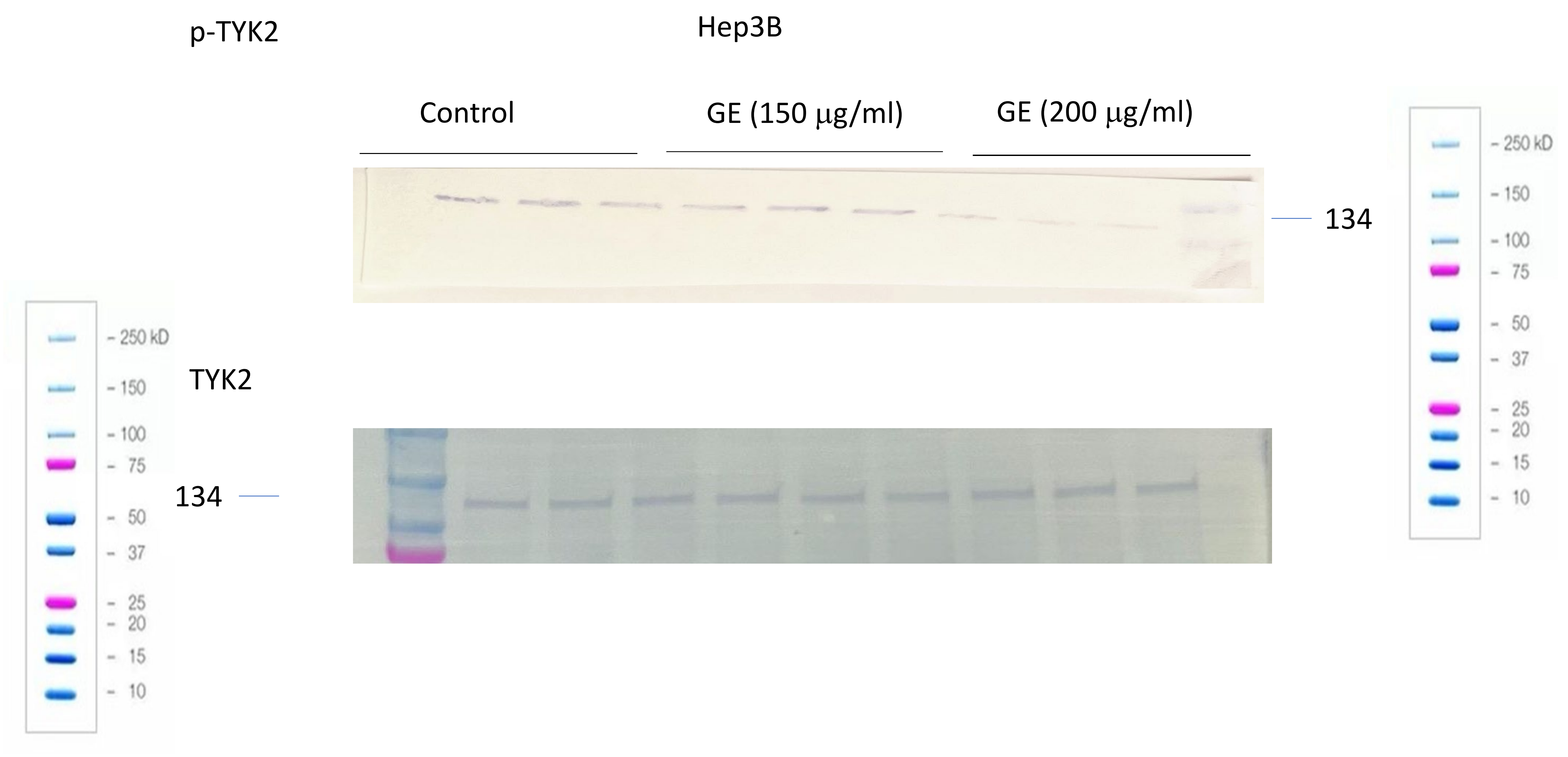
p-TYK2
Hep3B
Control
GE (150 μg/ml)
GE (200 μg/ml)
134
TYK2
134
250 kD
150
100
75
50
37
25
20
15
10
250 kD
150
100
75
50
37
25
20
15
10

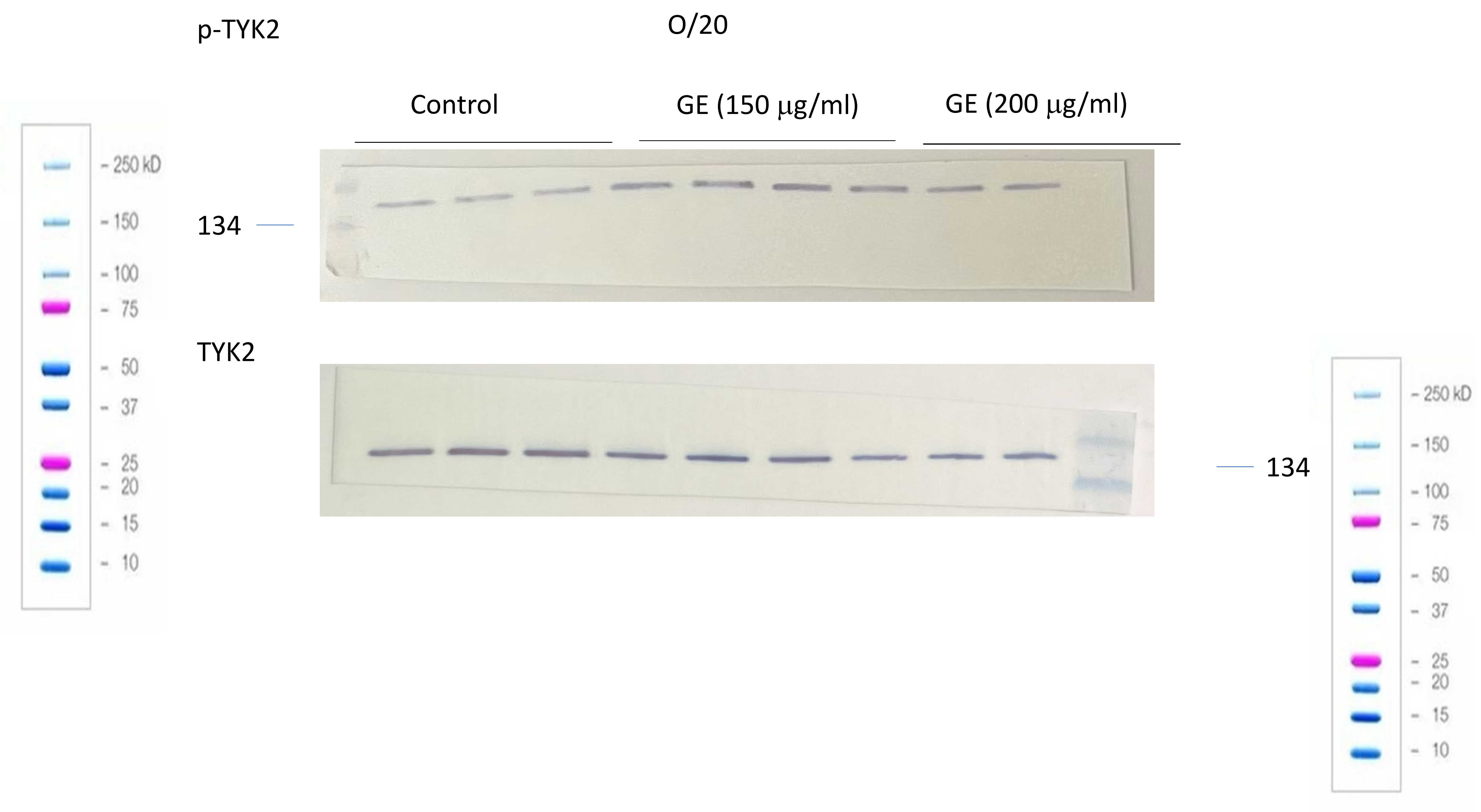

p-TYK2
O/20
Control
GE (150 μg/ml)
GE (200 μg/ml)
134
TYK2
134
250 kD
150
100
75
50
37
25
20
15
10

JAK1

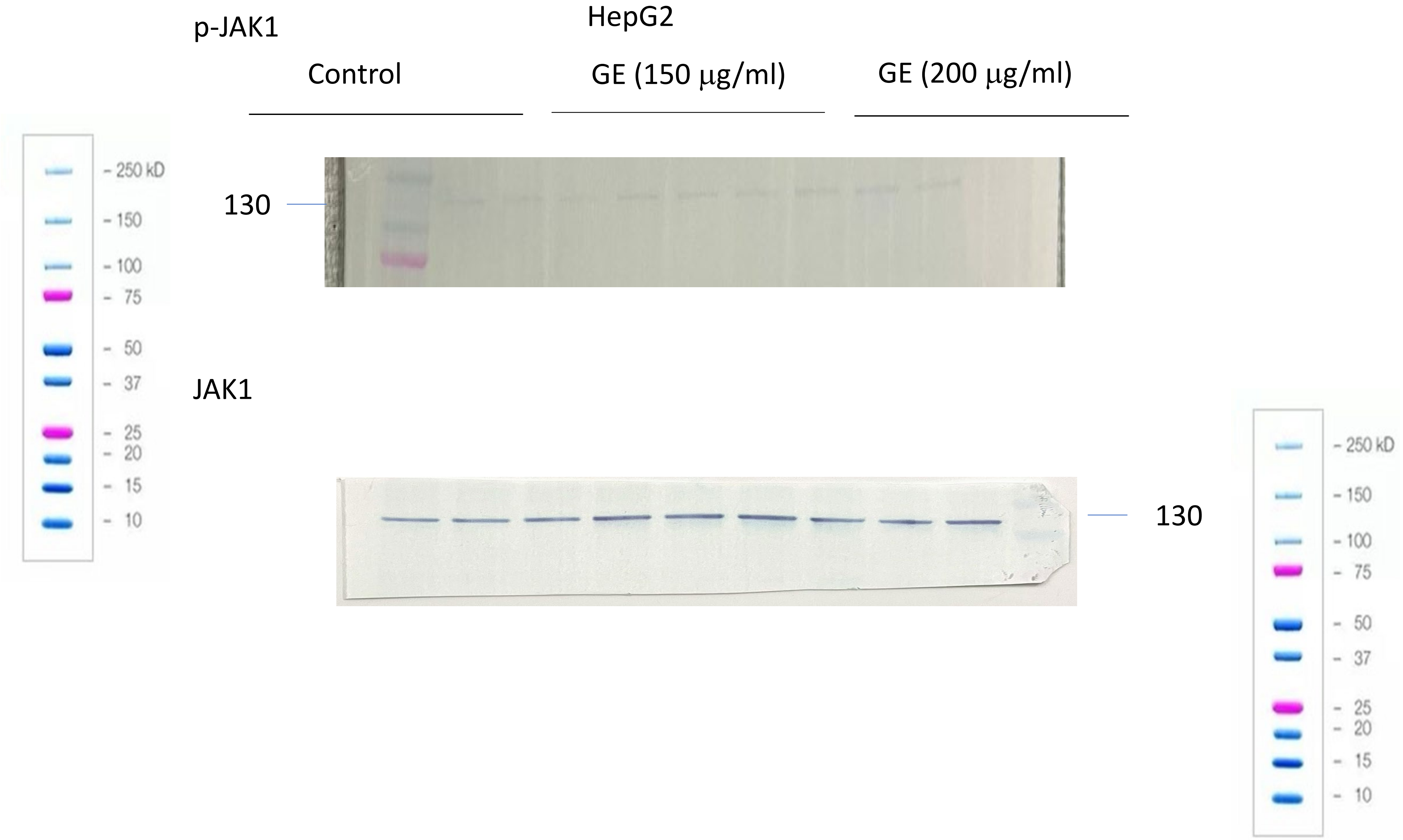
p-JAK1
HepG2
Control
GE (150 μg/ml)
GE (200 μg/ml)
130
JAK1
130
250 kD
150
100
75
50
37
25
20
15
10
250 kD
150
100
75
50
37
25
20
15
10

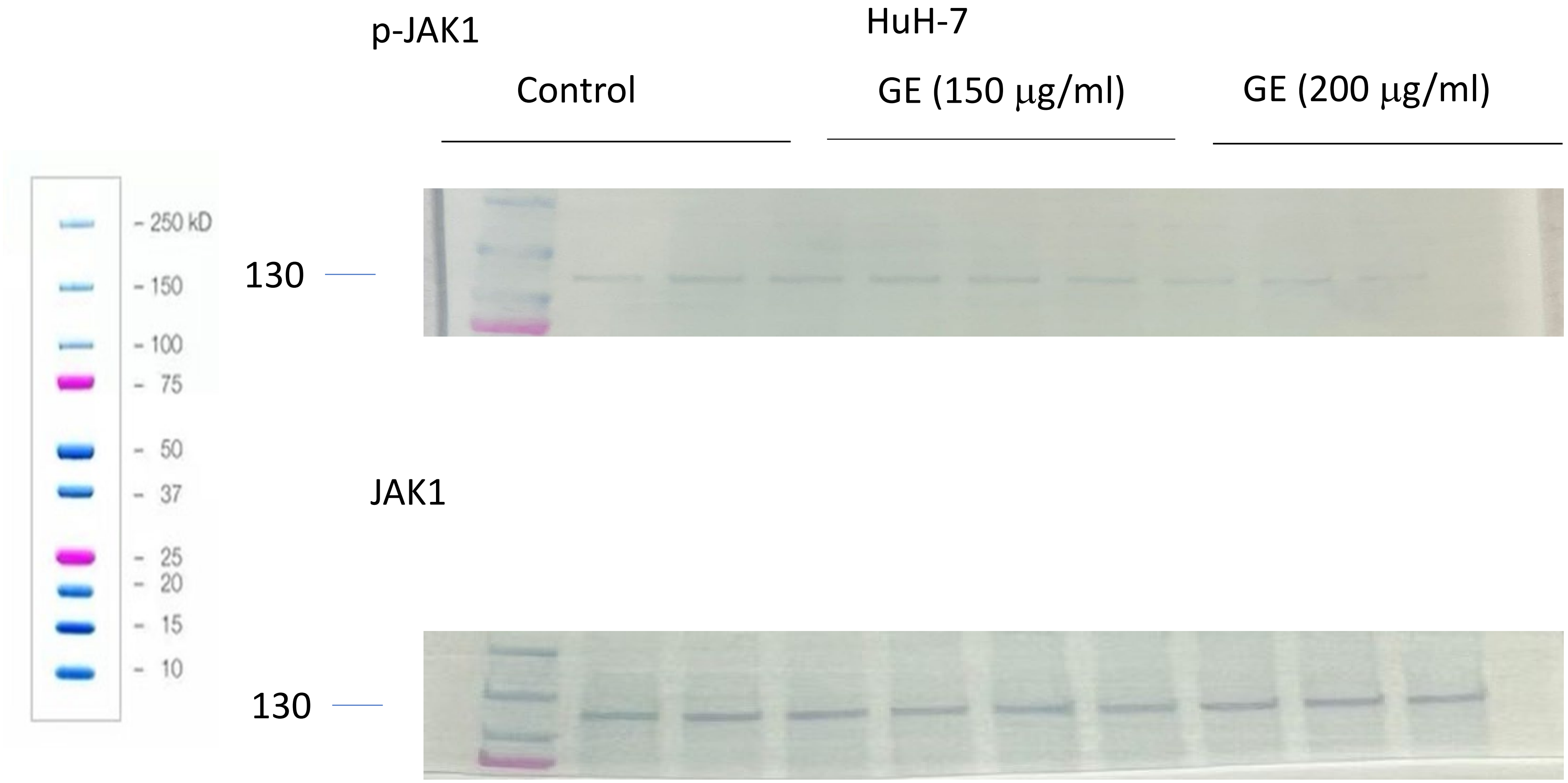
p-JAK1
HuH-7
Control
GE (150 μg/ml)
GE (200 μg/ml)
250 kD
150
100
75
50
37
25
20
15
10
130
JAK1
130

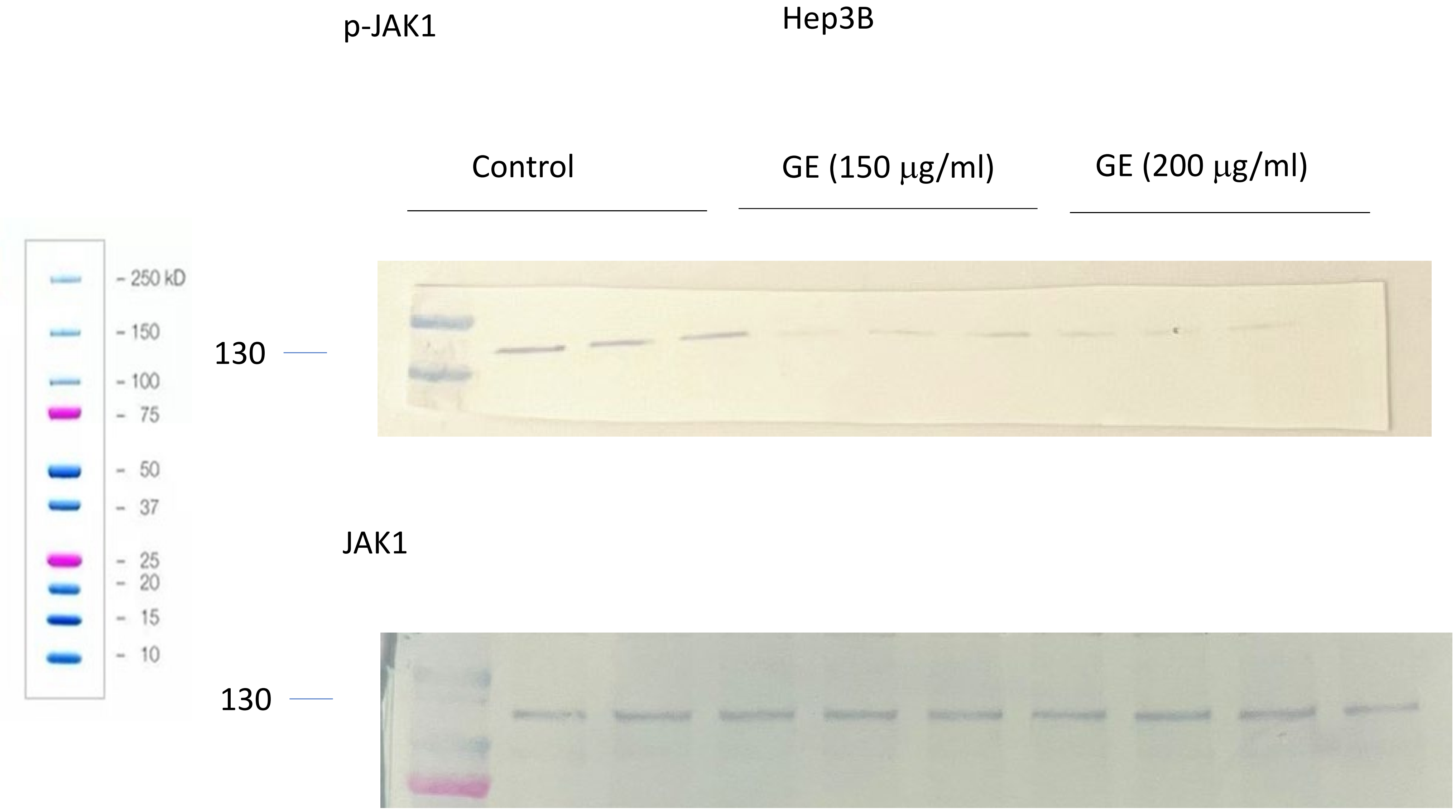
p-JAK1
Hep3B
Control
GE (150 μg/ml)
GE (200 μg/ml)
250 kD
150
100
75
50
37
25
20
15
10
130
JAK1
130

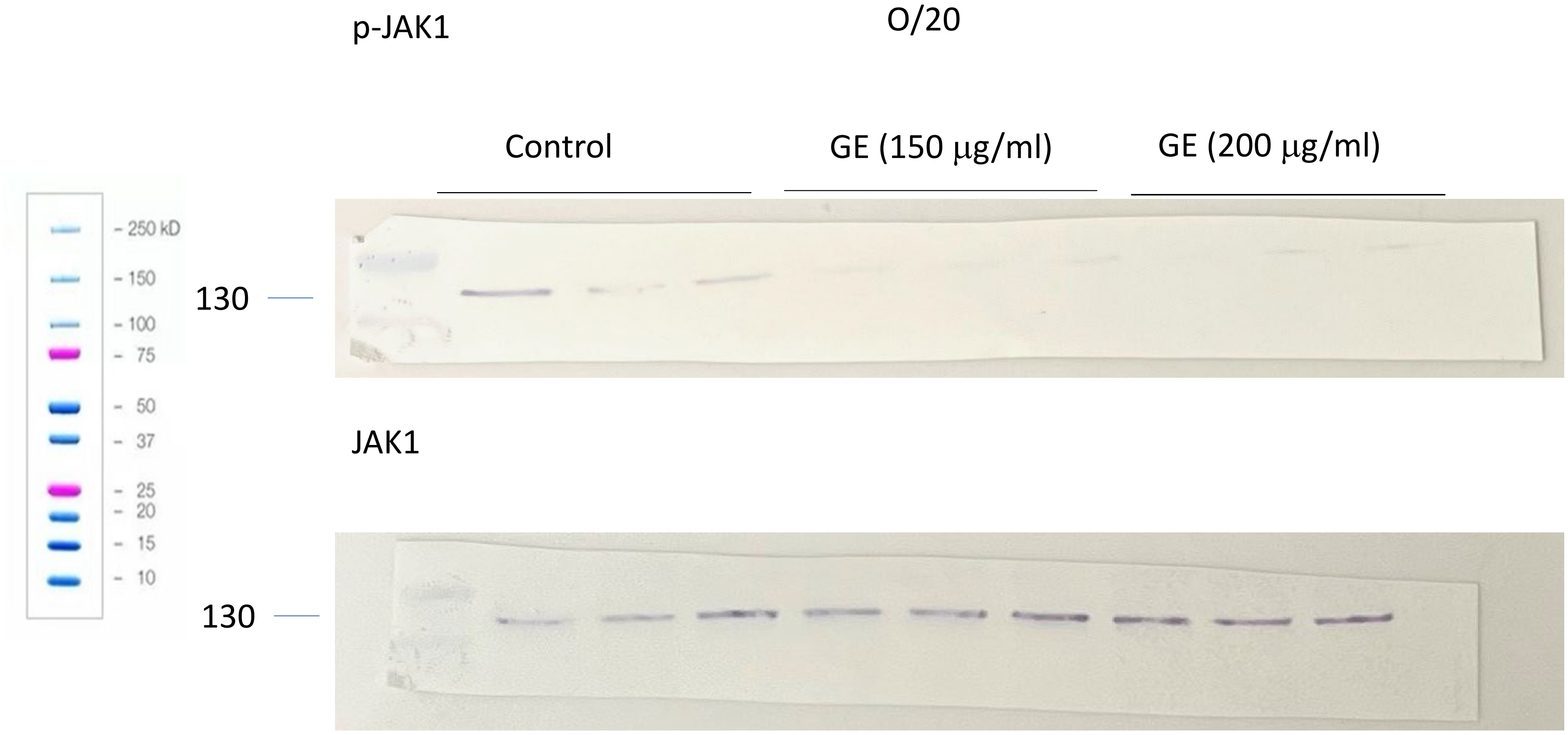
p-JAK1
O/20
Control
GE (150 μg/ml)
GE (200 μg/ml)
250 kD
150
100
75
50
37
25
20
15
10
130
JAK1
130

STAT 2

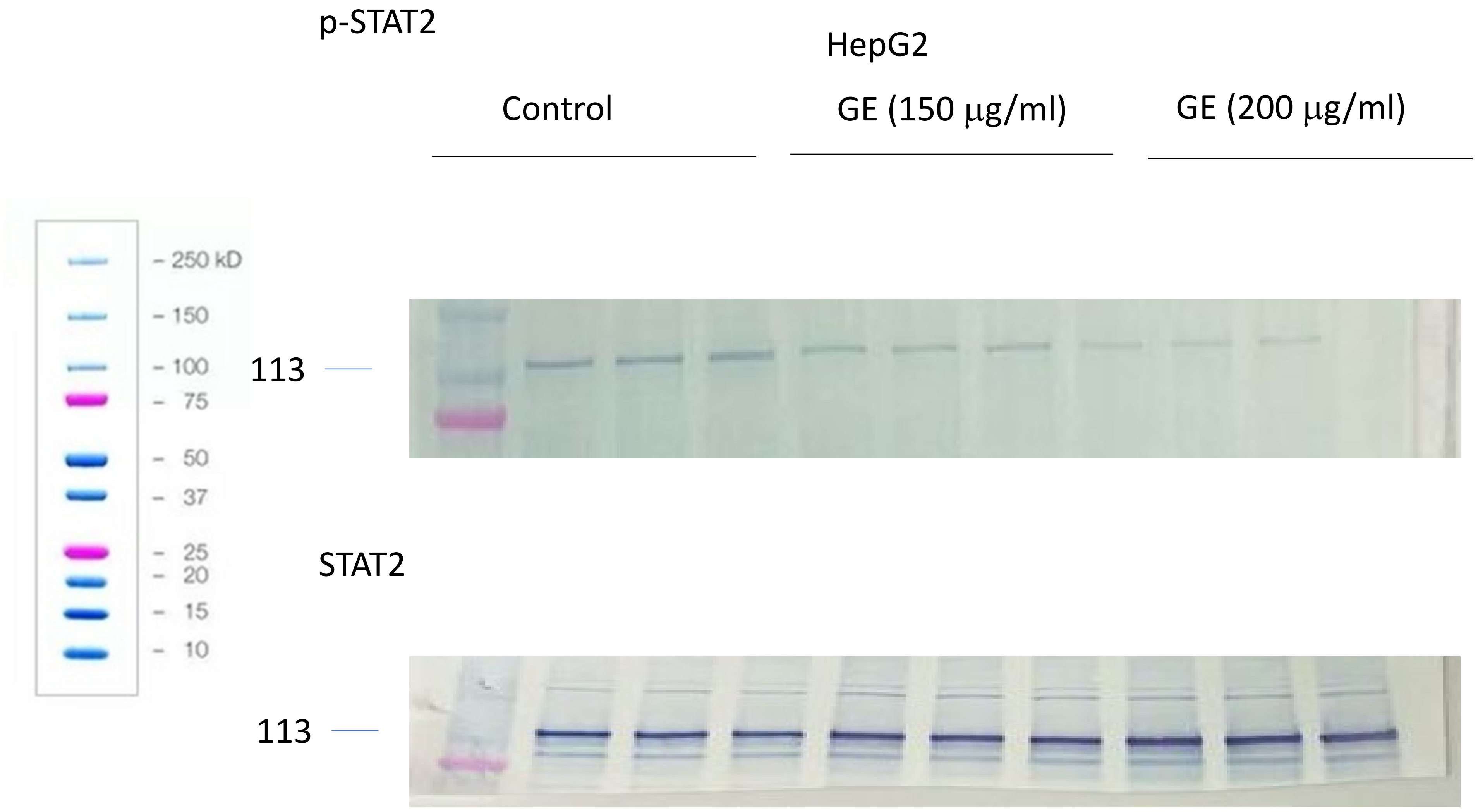
p-STAT2
HepG2
Control
GE (150 μg/ml)
GE (200 μg/ml)
250 kD
150
100
75
50
37
25
20
15
10
113
STAT2
113

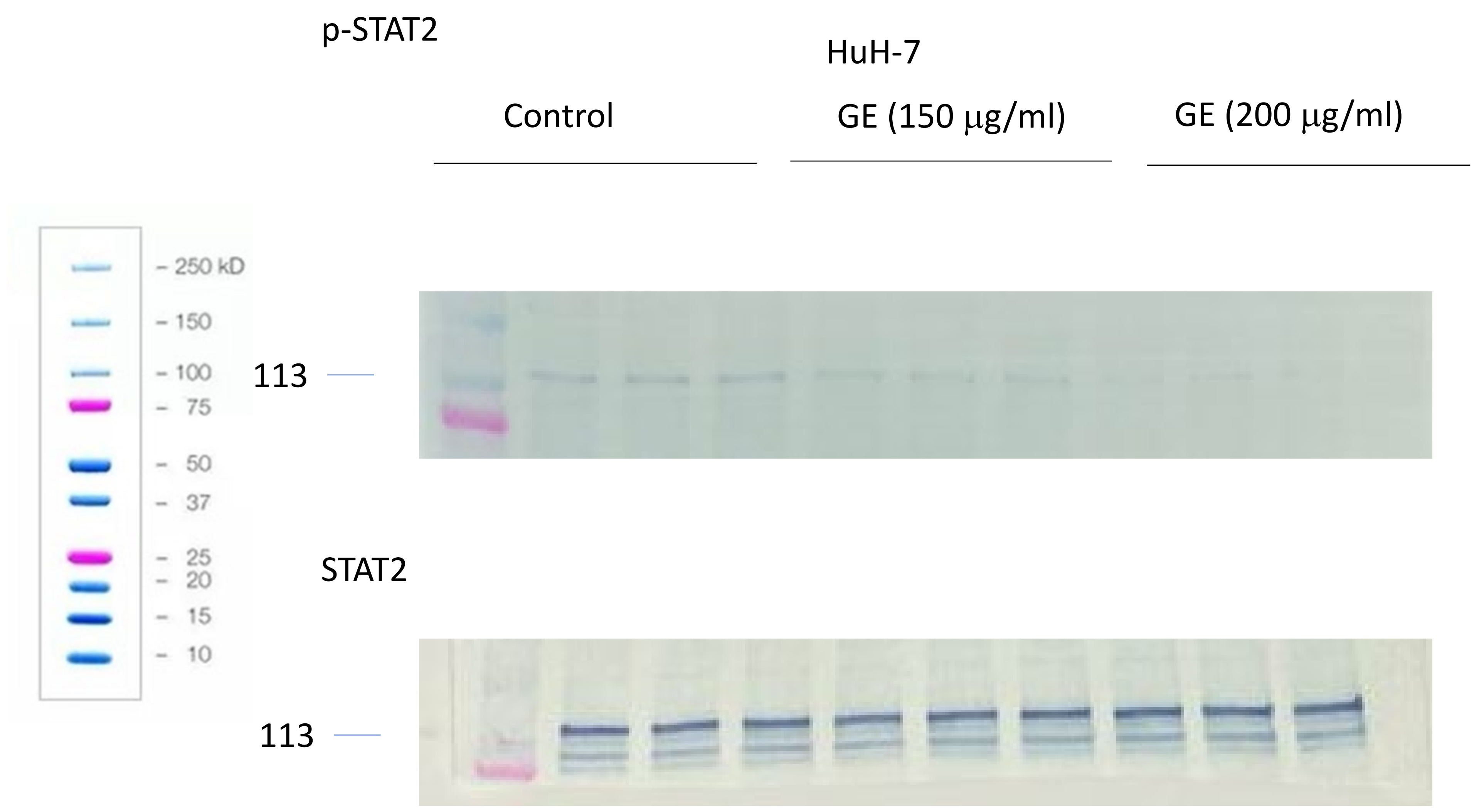
p-STAT2
HuH-7
Control
GE (150 μg/ml)
GE (200 μg/ml)
250 kD
150
100
75
50
37
25
20
15
10
113
STAT2
113

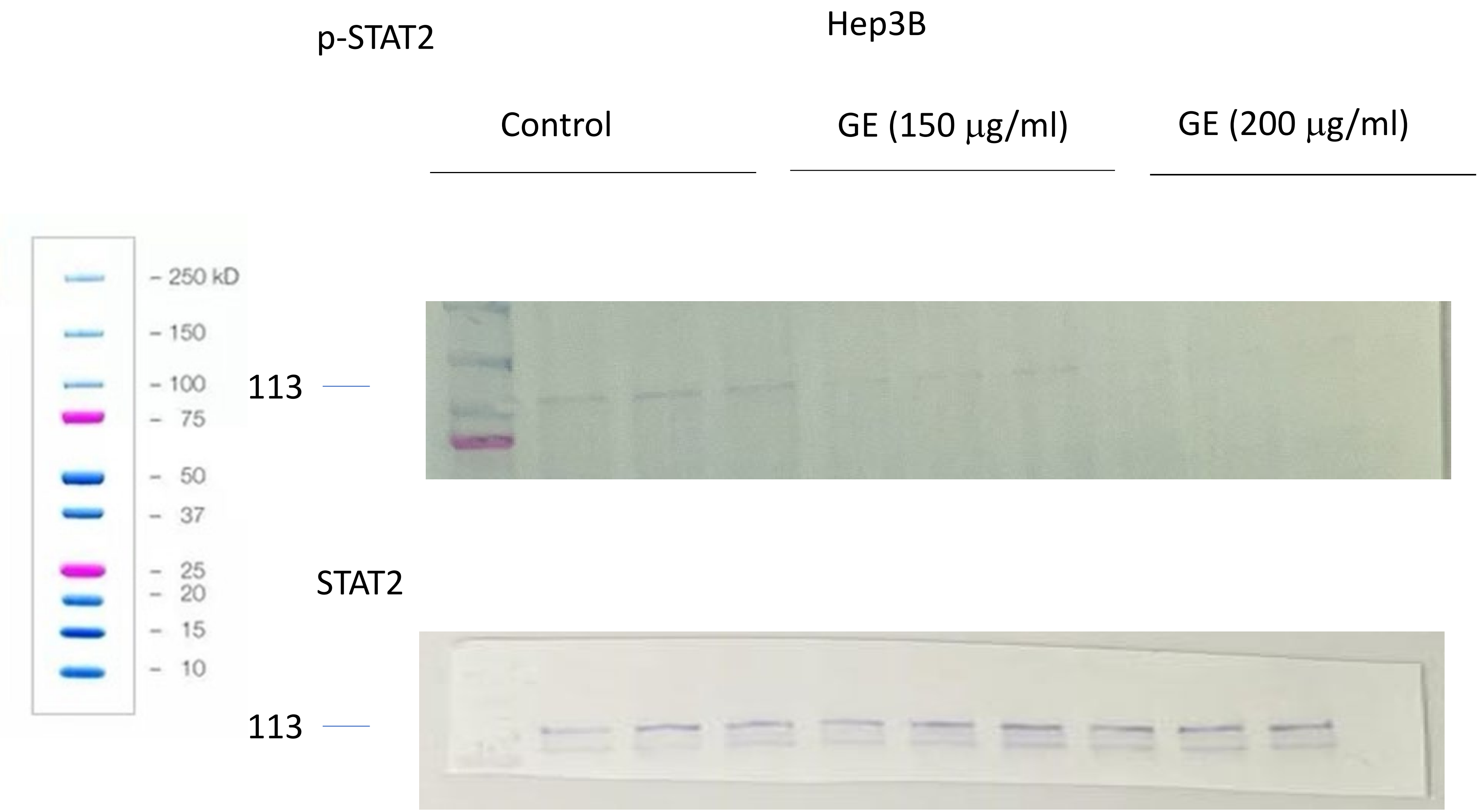

p-STAT2
Hep3B
Control
GE (150 μg/ml)
GE (200 μg/ml)
250 kD
150
100
75
50
37
25
20
15
10
113
STAT2
113

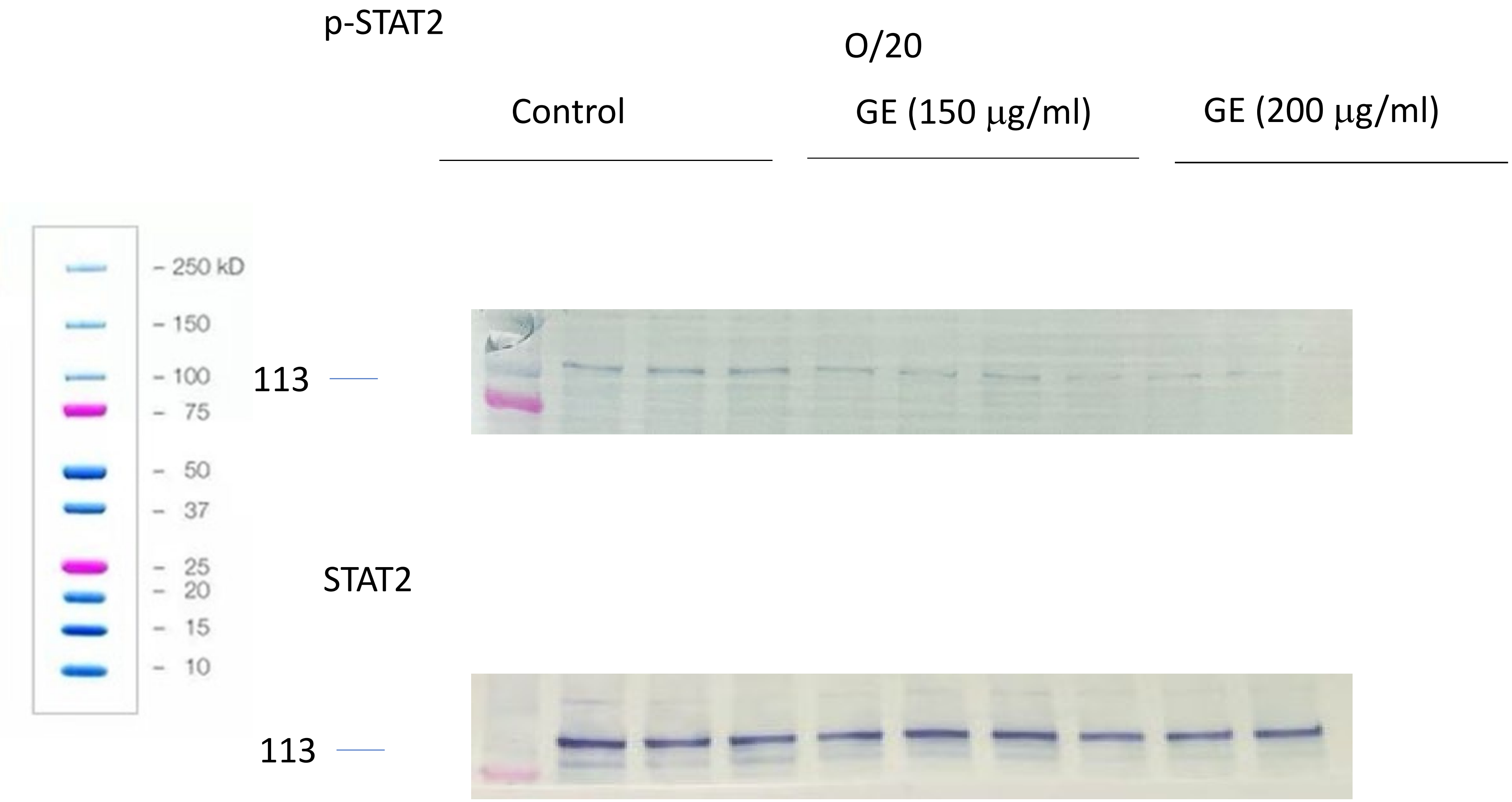
p-STAT2
O/20
Control
GE (150 μg/ml)
GE (200 μg/ml)
250 kD
150
100
75
50
37
25
20
15
10
113
STAT2
113

STAT 1

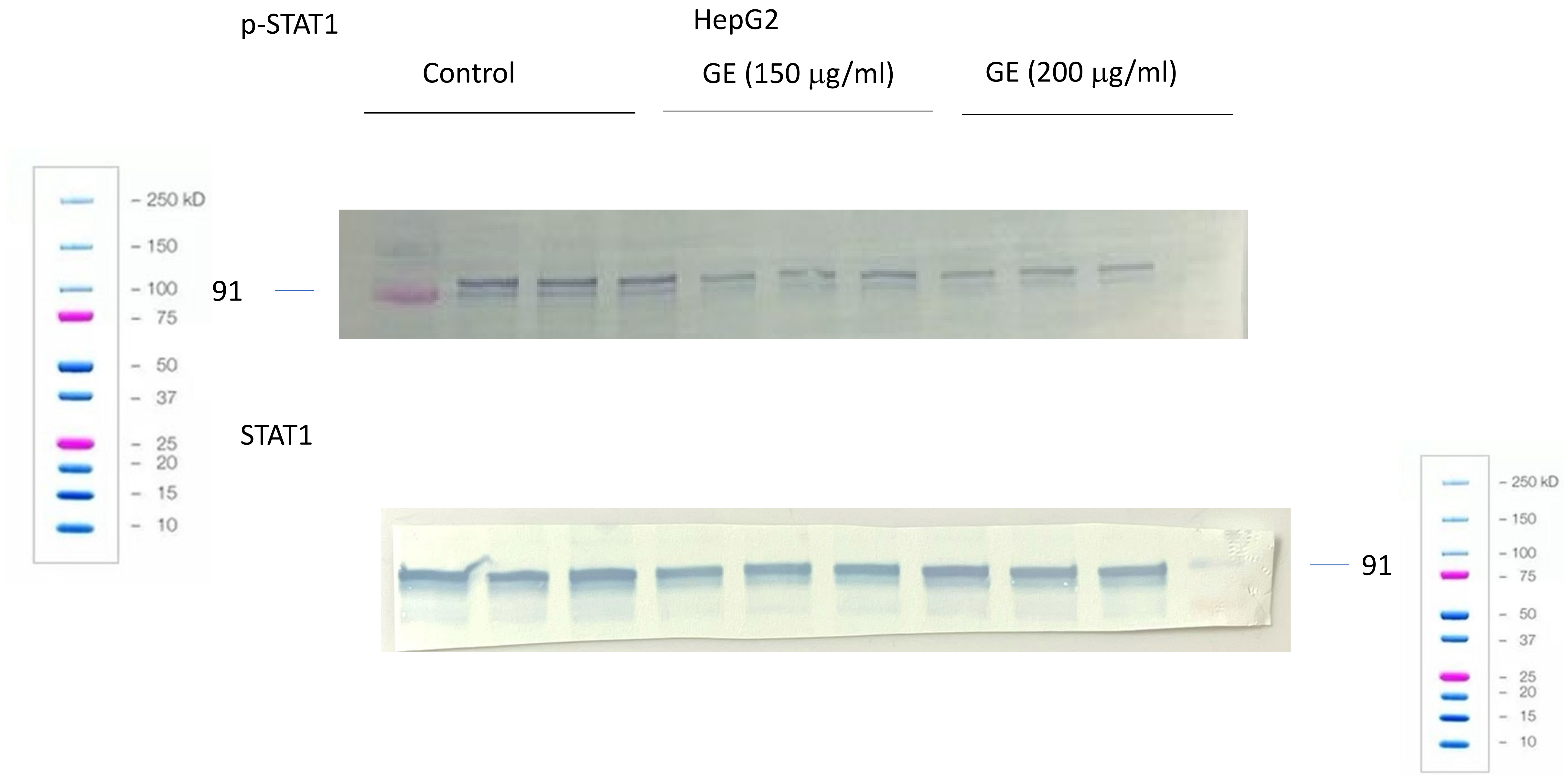
p-STAT1
HepG2
Control
GE (150 μg/ml)
GE (200 μg/ml)
250 kD
150
100
75
50
37
25
20
15
10
91
STAT1
91
250 kD
150
100
75
50
37
25
20
15
10

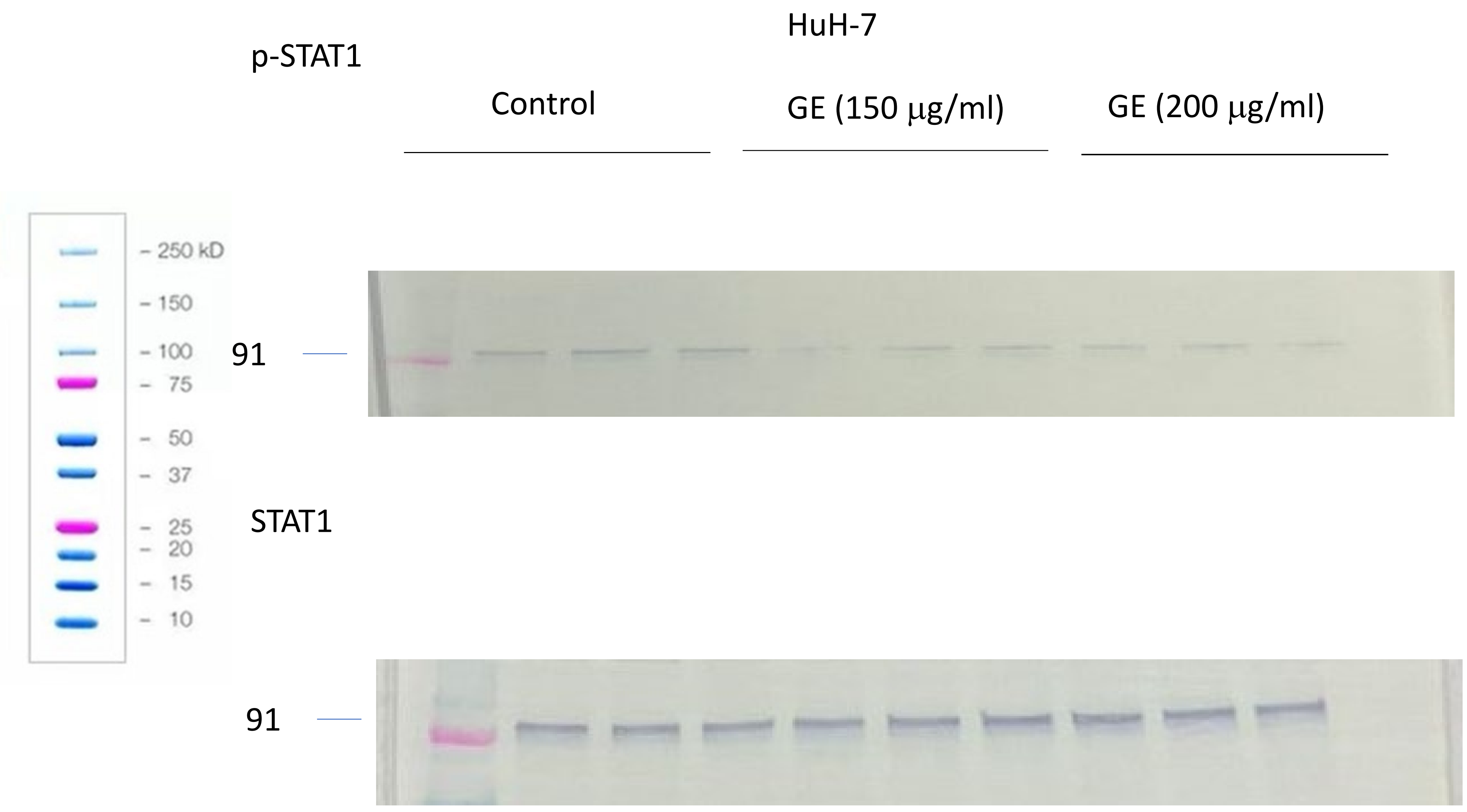
HuH-7
p-STAT1
Control
GE (150 μg/ml)
GE (200 μg/ml)
250 kD
150
100
75
50
37
25
20
15
10
91
STAT1
91

p-STAT1

Hep3B

Control GE (150 μg/ml) GE (200 μg/ml)

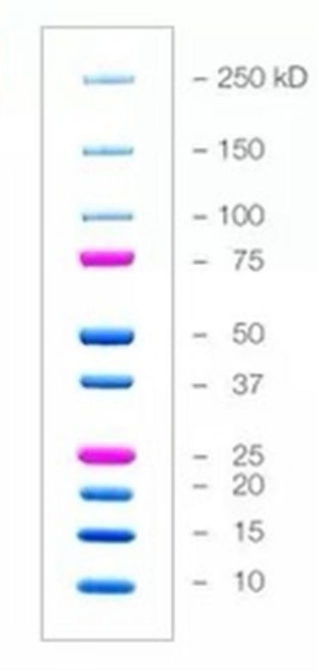

91

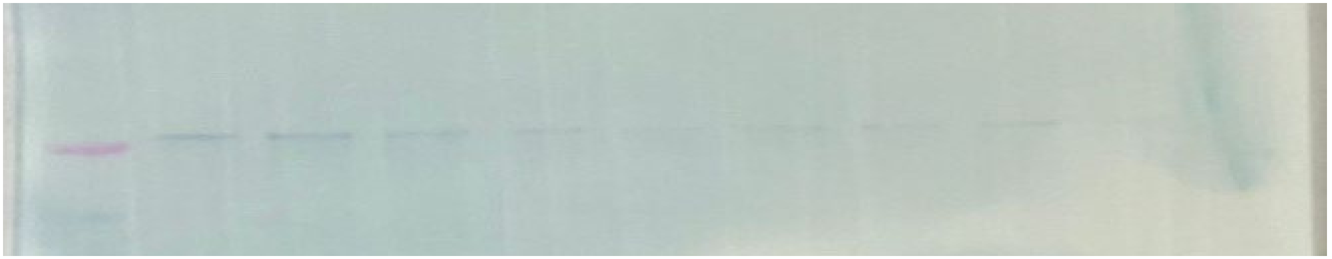

STAT1

91

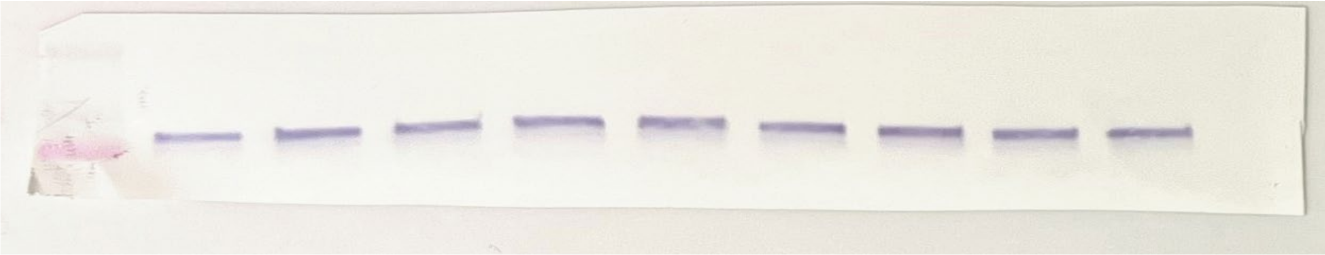

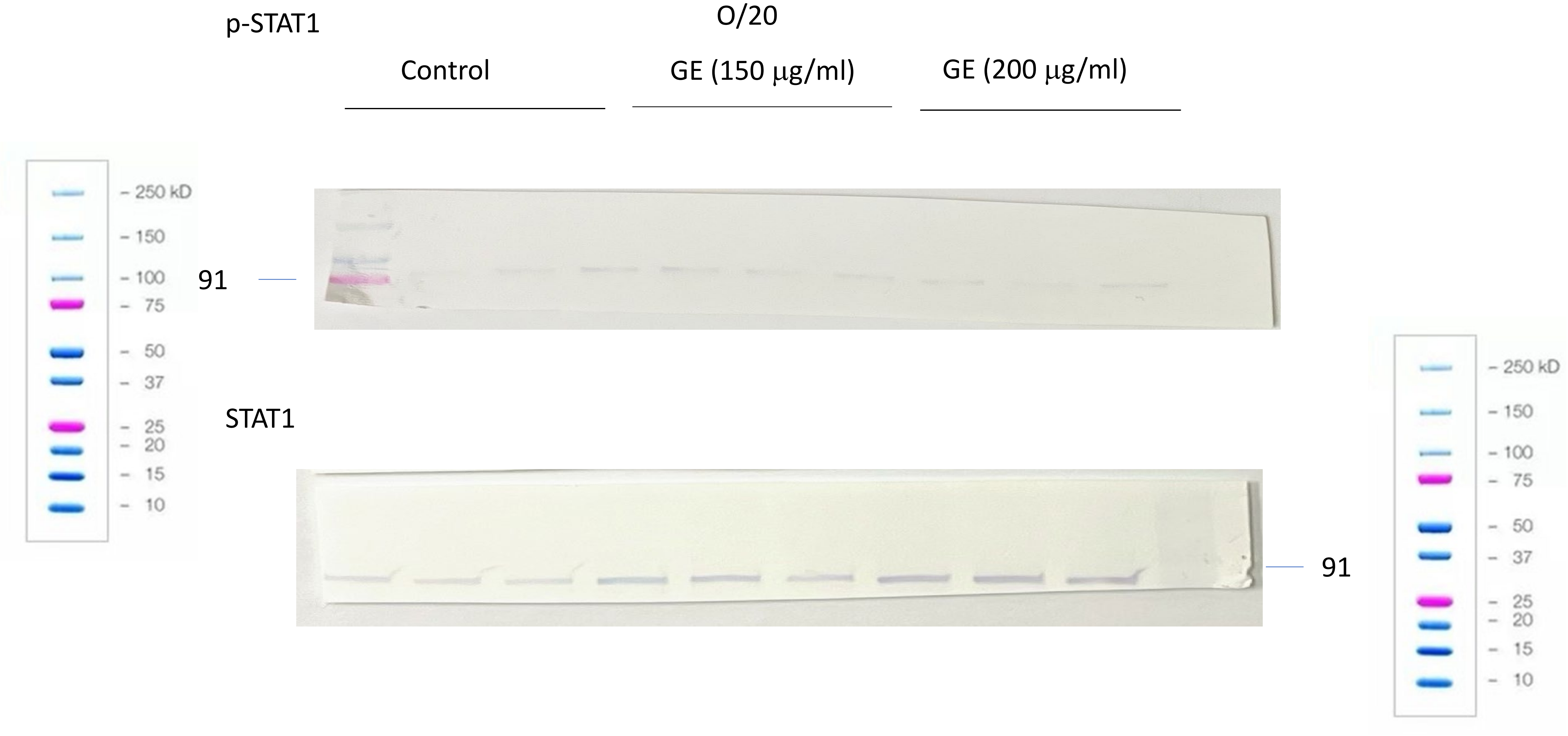
p-STAT1
O/20
Control
GE (150 μg/ml)
GE (200 μg/ml)
91
STAT1
91
250 kD
150
100
75
50
37
25
20
15
10

Beta Actinj HepG2

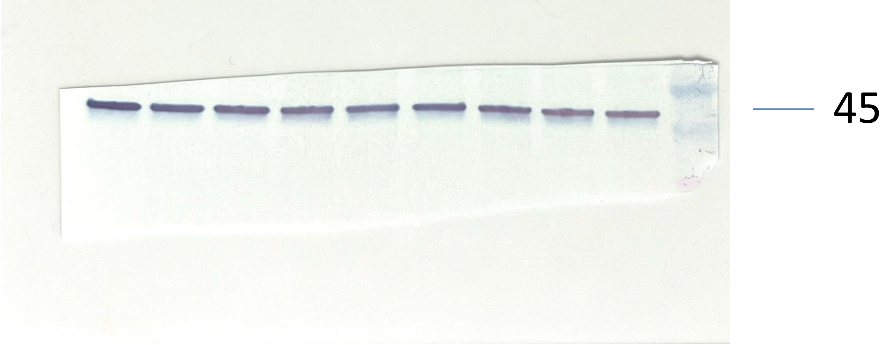

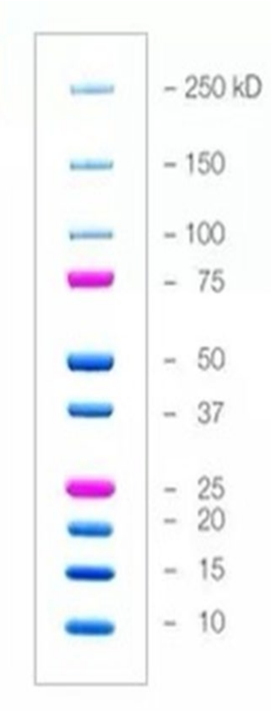

Beta Actin HuH-7

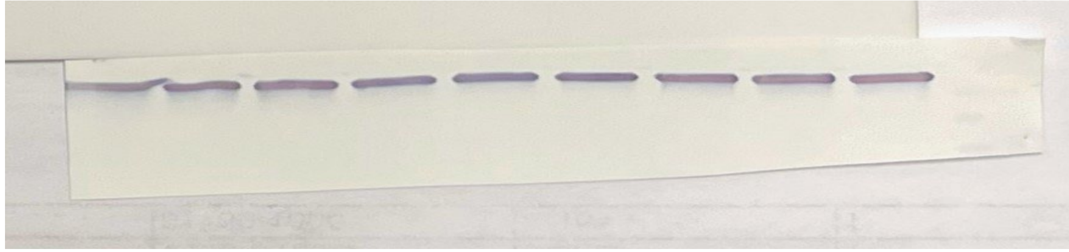

45

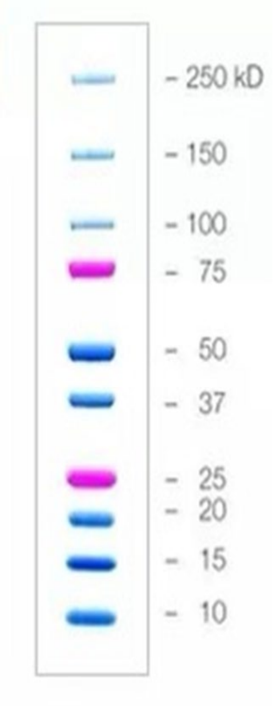

Beta Actin Hep3B

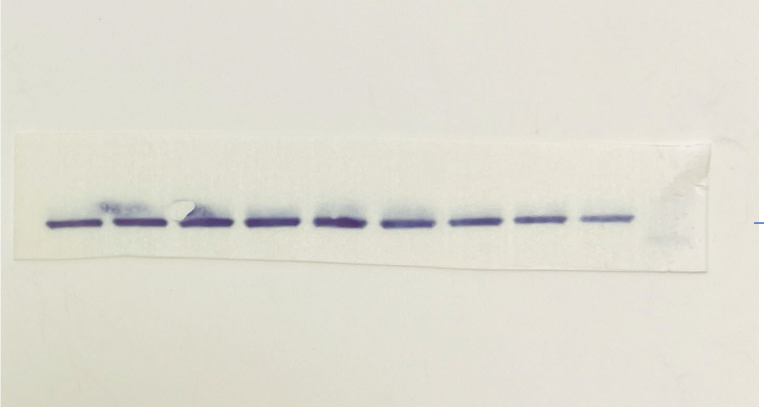

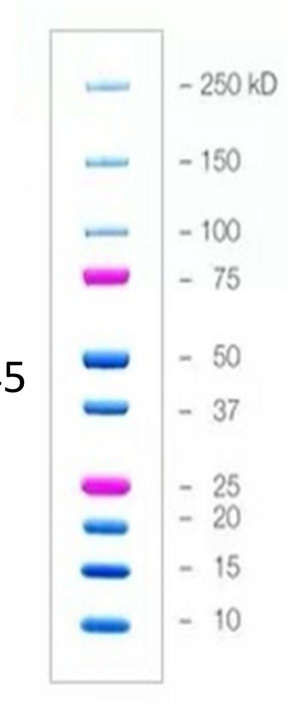

Beta Actin O/20

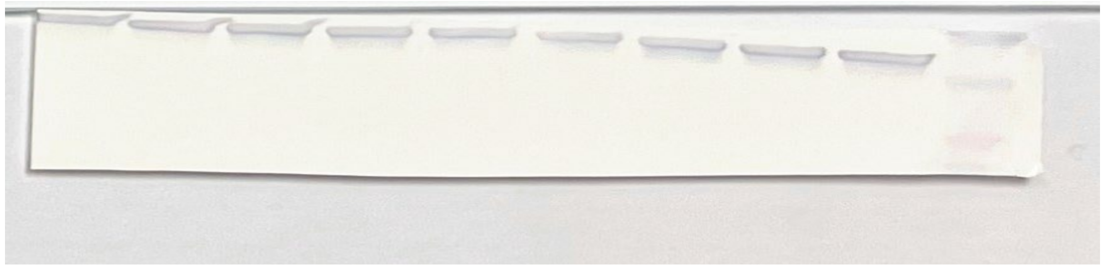

45

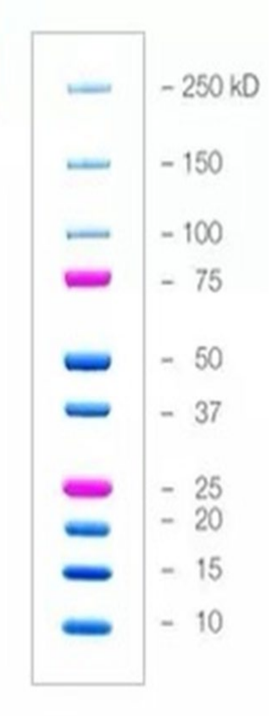

Supplement: Supplementary file 1 [file cancers-15-04283-s001.zip › File S1.pdf]
