# Supplementary material for: Dysregulation of Type I Interferon (IFN-I) Signaling: A Potential Contributor to Racial Disparity in Hepatocellular Carcinoma (HCC)
Source: Cancers (Basel). 2023 Aug 27;15(17):4283. doi: 10.3390/cancers15174283 (PMC10486472; doi:10.3390/cancers15174283)
Supplement: Supplementary file 1 [file cancers-15-04283-s001.zip › Supplementary Tables S1 and S2.pdf]

Table S1. Differentially expressed genes between 17 AA/Black and 184 White HCC samples from TCGA. "logFC" - log2 fold-change, "AveExpr" - log2 average expression, "P.Value" and "adj.P.Val" - raw and FDR-adjusted p-values.

Gene	logFC	AveExpr	P.Value	adj.P.Val
ARSE	-6.009	5.521	3.18E-17	6.52E-13
LOC90784	-1.272	3.005	8.81E-09	9.03E-05
GAGE13	1.983	-5.322	5.98E-07	4.09E-03
MS4A8B	2.902	-4.504	9.88E-07	5.06E-03
GAGE2E	2.190	-5.307	2.06E-06	8.44E-03
LRRC37A2	-1.445	1.173	2.61E-06	8.93E-03
OR5H6	1.166	-5.433	3.40E-06	9.46E-03
MAGEB3	0.916	-5.573	3.69E-06	9.46E-03
EPB49	-1.358	4.183	4.35E-06	9.91E-03
CRYBB2	1.630	-3.818	5.23E-06	1.07E-02
GAGE1	3.348	-4.428	6.21E-06	1.16E-02
GUCA1B	-1.411	-0.482	1.07E-05	1.83E-02
RNF17	2.678	-4.626	1.84E-05	2.90E-02
CYP3A5	1.693	6.724	2.00E-05	2.90E-02
C22orf13	-0.749	7.308	2.12E-05	2.90E-02
IFI35	0.960	5.096	2.35E-05	3.01E-02
POLR1A	0.812	4.132	3.56E-05	4.30E-02
GAGE12J	2.986	-4.169	5.25E-05	5.98E-02
GAGE4	3.481	-3.764	6.17E-05	6.49E-02
PASD1	1.253	-5.527	6.33E-05	6.49E-02
HEPHL1	1.379	-4.480	6.92E-05	6.75E-02
GAGE2D	3.611	-3.778	9.54E-05	8.23E-02
C17orf46	1.145	-4.590	9.61E-05	8.23E-02
GAGE12D	3.565	-3.843	9.92E-05	8.23E-02
PRPS1	0.585	5.329	1.10E-04	8.23E-02
CSAG3	3.713	-2.010	1.11E-04	8.23E-02
NXF2	2.832	-4.491	1.12E-04	8.23E-02
TTLL9	-1.032	-3.000	1.12E-04	8.23E-02
MAGEA11	2.329	-4.543	1.22E-04	8.66E-02
ACSM1	3.099	2.659	1.29E-04	8.78E-02
CRISP2	1.794	-4.971	1.58E-04	1.05E-01
GUSBP3	-1.370	0.533	1.67E-04	1.07E-01
LOC148824	1.433	-4.792	1.86E-04	1.13E-01
PAGE1	3.104	-4.108	1.92E-04	1.13E-01
UTP6	0.563	4.736	1.96E-04	1.13E-01
ADRA2B	-1.081	0.381	1.98E-04	1.13E-01
CPXCR1	0.676	-5.585	2.34E-04	1.29E-01
LOC400931	-1.105	-1.610	2.41E-04	1.30E-01

NCRNA00200	1.910	-4.857	2.85E-04	1.42E-01
OR2C3	0.910	-5.502	2.94E-04	1.42E-01
CCDC144NL	1.654	-4.915	2.95E-04	1.42E-01
PSG7	0.944	-5.422	2.96E-04	1.42E-01
PRSS45	1.637	-3.361	2.97E-04	1.42E-01
MAGEB2	3.341	-3.274	3.12E-04	1.45E-01
SPAG6	1.311	-4.854	3.67E-04	1.63E-01
LAIR2	1.515	-3.894	3.72E-04	1.63E-01
CDK11B	0.508	4.718	3.81E-04	1.63E-01
C7orf33	0.752	-5.416	3.88E-04	1.63E-01
HERC2P2	-1.010	4.271	3.90E-04	1.63E-01
FAM183A	1.503	-4.679	3.98E-04	1.63E-01
ZNF90	0.966	1.554	4.43E-04	1.78E-01
PSMD2	0.477	7.141	4.53E-04	1.79E-01
LOC440173	2.131	-3.922	5.69E-04	2.17E-01
CXorf49B	1.554	-5.105	5.73E-04	2.17E-01
GAGE2A	2.320	-4.548	6.08E-04	2.26E-01
ARSG	-1.151	1.033	6.27E-04	2.26E-01
ISG15	1.239	5.438	6.29E-04	2.26E-01
CSAG1	3.152	-2.549	6.70E-04	2.37E-01
CNGB3	1.665	-4.603	6.88E-04	2.39E-01
CLEC17A	1.094	-4.760	7.07E-04	2.41E-01
ATAD3C	1.673	0.239	7.37E-04	2.48E-01
DNAJB12	-0.366	5.500	7.65E-04	2.53E-01
SUMF2	-0.539	7.274	7.91E-04	2.58E-01
RPH3AL	-0.683	3.457	8.63E-04	2.73E-01
EDARADD	1.270	0.529	8.71E-04	2.73E-01
FAM3A	0.522	5.807	8.80E-04	2.73E-01
SAR1A	-0.352	6.377	9.24E-04	2.83E-01
MX1	1.185	5.301	9.54E-04	2.85E-01
TENC1	-0.642	6.133	9.58E-04	2.85E-01
NUDT13	-0.921	1.870	9.77E-04	2.86E-01
MAD2L2	0.599	4.794	1.02E-03	2.94E-01
TCL1B	1.060	-5.184	1.07E-03	3.01E-01
TSPY3	1.853	-5.068	1.08E-03	3.01E-01
SYCP1	0.527	-5.726	1.09E-03	3.01E-01
NXF5	1.163	-4.972	1.11E-03	3.02E-01
FLJ25363	0.912	-5.389	1.12E-03	3.02E-01
TAP2	0.761	5.639	1.13E-03	3.02E-01
PXDNL	-1.326	-1.607	1.16E-03	3.04E-01
CT45A6	0.843	-5.620	1.17E-03	3.04E-01
ERAP1	0.754	5.726	1.19E-03	3.05E-01
RSAD2	1.152	1.609	1.28E-03	3.24E-01

MAGEA4	1.841	-4.949	1.33E-03	3.33E-01
FCRL4	0.760	-5.468	1.38E-03	3.34E-01
ERRFI1	-1.123	7.749	1.38E-03	3.34E-01
ADRB1	-1.455	-2.202	1.39E-03	3.34E-01
MYT1L	1.449	-5.167	1.50E-03	3.57E-01
NOTCH2NL	0.793	4.268	1.56E-03	3.59E-01
IFI27	1.988	6.059	1.56E-03	3.59E-01
CXorf48	1.717	-4.694	1.57E-03	3.59E-01
TSPY4	1.391	-5.390	1.58E-03	3.59E-01
CCL8	1.262	-0.770	1.59E-03	3.59E-01
IFI44L	1.455	2.016	1.62E-03	3.60E-01
C21orf121	-1.533	-4.240	1.67E-03	3.69E-01
LQK1	-1.259	0.820	1.72E-03	3.69E-01
AVEN	-0.476	3.437	1.74E-03	3.69E-01
CSMD3	1.608	-4.669	1.74E-03	3.69E-01
KHDC1L	1.434	-5.103	1.79E-03	3.69E-01
MAGEE2	0.891	-5.285	1.79E-03	3.69E-01
OPRK1	1.148	-5.184	1.79E-03	3.69E-01
BLK	1.471	-2.735	1.81E-03	3.69E-01
BCR	-0.506	5.531	1.83E-03	3.69E-01
OAS3	1.020	4.544	1.83E-03	3.69E-01
AGAP7	-0.900	-1.606	1.88E-03	3.73E-01
PSMA6	0.371	6.541	2.01E-03	3.95E-01
AQPEP	1.364	-3.541	2.13E-03	4.16E-01
ABCD4	-0.442	4.812	2.15E-03	4.16E-01
EP400NL	-0.653	1.986	2.21E-03	4.21E-01
OBFC1	-0.462	3.677	2.22E-03	4.21E-01
EYA4	2.052	-3.778	2.26E-03	4.26E-01
CXorf61	1.537	-5.011	2.34E-03	4.37E-01
KY	1.201	-4.539	2.39E-03	4.42E-01
GRID2	0.589	-5.586	2.43E-03	4.45E-01
IFI6	1.240	6.102	2.47E-03	4.48E-01
NAA11	2.384	-3.913	2.52E-03	4.54E-01
PPP2R2D	-0.367	4.147	2.58E-03	4.59E-01
IL28B	0.255	-5.807	2.60E-03	4.59E-01
CECR7	2.020	-3.439	2.63E-03	4.61E-01
SORBS1	-0.666	5.449	2.68E-03	4.62E-01
BMS1P4	-0.889	-0.942	2.68E-03	4.62E-01
TM4SF20	3.193	-1.829	2.74E-03	4.68E-01
MAP3K14	0.824	4.376	2.87E-03	4.79E-01
ADAM20	-0.747	-4.677	2.87E-03	4.79E-01
CATSPER2	-0.723	-0.630	2.88E-03	4.79E-01
FAM133B	-0.561	1.329	2.90E-03	4.79E-01

SCGB3A2	0.555	-5.583	2.96E-03	4.83E-01
NLRP4	1.497	-4.928	2.97E-03	4.83E-01
GAGE8	1.472	-5.055	3.06E-03	4.94E-01
FAM182A	1.112	-4.646	3.19E-03	5.12E-01
GUCY2C	2.689	-1.445	3.32E-03	5.19E-01
MINA	0.658	4.682	3.32E-03	5.19E-01
PDE9A	-1.622	1.689	3.33E-03	5.19E-01
VCY	1.967	-4.727	3.34E-03	5.19E-01
LARGE	-0.599	4.110	3.37E-03	5.20E-01
NXF2B	0.844	-5.523	3.40E-03	5.20E-01
CSTF2	0.593	2.987	3.46E-03	5.25E-01
VGLL2	1.095	-5.407	3.62E-03	5.45E-01
HLA.DOB	1.389	0.001	3.69E-03	5.46E-01
H1FNT	1.206	-4.835	3.70E-03	5.46E-01
MRPL42P5	-1.037	-2.544	3.71E-03	5.46E-01
PANX3	0.837	-5.314	3.73E-03	5.46E-01
CCL3L1	1.372	-1.057	3.76E-03	5.46E-01
D2HGDH	-0.480	4.861	3.80E-03	5.49E-01
SPATA20	-0.620	5.488	3.88E-03	5.56E-01
SEC1	-0.880	-3.412	3.92E-03	5.58E-01
PJA1	0.623	3.532	4.02E-03	5.68E-01
PRPS1L1	0.928	-4.998	4.04E-03	5.68E-01
C17orf66	0.826	-4.939	4.19E-03	5.84E-01
CYB5D2	-0.590	4.312	4.28E-03	5.93E-01
PCDHB19P	-1.008	-3.784	4.32E-03	5.94E-01
PSMA8	1.131	-5.094	4.35E-03	5.94E-01
PSMB9	0.882	4.755	4.48E-03	6.07E-01
TNP1	1.075	-5.383	4.50E-03	6.07E-01
TLR9	0.926	-0.992	4.58E-03	6.07E-01
NCRNA00105	-0.950	1.533	4.62E-03	6.07E-01
CASP9	0.560	3.592	4.63E-03	6.07E-01
GAGE2B	2.158	-4.687	4.63E-03	6.07E-01
TREX1	0.779	3.088	4.65E-03	6.07E-01
CD1B	1.322	-3.689	4.68E-03	6.08E-01
MAGEA8	2.014	-4.126	4.76E-03	6.12E-01
LY6G5C	-0.763	-0.168	4.78E-03	6.12E-01
EGFLAM	-0.757	1.567	4.81E-03	6.13E-01
CMPK2	0.992	2.258	4.85E-03	6.13E-01
RIPPLY2	0.985	-5.320	4.95E-03	6.18E-01
LAS1L	0.384	5.313	4.96E-03	6.18E-01
TTC29	0.539	-5.605	5.01E-03	6.18E-01
POM121L8P	-0.778	-4.778	5.01E-03	6.18E-01
OR52N2	0.643	-5.371	5.04E-03	6.19E-01

SOX30	0.862	-4.576	5.13E-03	6.26E-01
PLXNA2	-0.551	4.459	5.26E-03	6.32E-01
C6orf52	-1.240	-2.772	5.26E-03	6.32E-01
COL4A6	1.730	-3.006	5.30E-03	6.32E-01
TNFRSF10D	-0.802	3.277	5.30E-03	6.32E-01
RPS27L	-0.463	6.125	5.38E-03	6.35E-01
C12orf63	0.869	-4.422	5.41E-03	6.35E-01
CCL4L2	1.165	0.478	5.43E-03	6.35E-01
PRMT5	0.430	5.117	5.46E-03	6.35E-01
HGS	0.450	6.271	5.48E-03	6.35E-01
POPDC3	1.862	-3.880	5.52E-03	6.35E-01
LOC285780	1.009	-5.090	5.55E-03	6.35E-01
HERC5	1.126	2.589	5.67E-03	6.45E-01
TOB2	-0.563	5.454	5.74E-03	6.49E-01
GAGE2C	1.321	-5.150	5.79E-03	6.49E-01
OAS1	0.782	4.755	5.81E-03	6.49E-01
PKP4	-0.479	5.694	5.84E-03	6.49E-01
SPATS1	0.805	-5.136	5.85E-03	6.49E-01
TCF7L2	-0.457	4.407	5.90E-03	6.50E-01
SLC25A16	-0.491	3.639	5.97E-03	6.54E-01
SUPT4H1	0.354	5.791	6.05E-03	6.54E-01
C3orf15	1.370	-0.389	6.12E-03	6.54E-01
PSMB2	0.353	6.610	6.14E-03	6.54E-01
PRSS50	1.535	-2.089	6.18E-03	6.54E-01
IFI44	1.036	3.433	6.18E-03	6.54E-01
NSF	0.436	5.077	6.21E-03	6.54E-01
EXOC2	0.441	4.586	6.29E-03	6.54E-01
PRR15L	-1.998	0.373	6.30E-03	6.54E-01
IL29	0.556	-5.428	6.37E-03	6.54E-01
MAGEA3	3.179	-1.685	6.38E-03	6.54E-01
GABRR3	1.060	-4.769	6.39E-03	6.54E-01
PFN3	0.462	-5.533	6.48E-03	6.54E-01
MAGEA6	3.335	-1.540	6.48E-03	6.54E-01
HESRG	0.587	-5.544	6.48E-03	6.54E-01
KIR3DL2	0.877	-4.623	6.55E-03	6.54E-01
TDRD7	0.488	3.701	6.57E-03	6.54E-01
BNC1	-1.093	-3.987	6.61E-03	6.54E-01
ADAM23	1.722	-2.269	6.64E-03	6.54E-01
TNFRSF13B	1.182	-3.852	6.66E-03	6.54E-01
TSPY2	1.707	-4.935	6.68E-03	6.54E-01
OR7C1	0.715	-5.549	6.81E-03	6.54E-01
C4orf50	0.535	-5.431	6.87E-03	6.54E-01
XAGE1D	3.645	-0.790	6.87E-03	6.54E-01

LRRIQ3	0.980	-2.264	6.90E-03	6.54E-01
ARMC4	1.453	-4.040	6.93E-03	6.54E-01
RNF215	-0.416	3.406	6.96E-03	6.54E-01
DHRS4	0.492	6.045	7.01E-03	6.54E-01
FAM46C	0.985	2.995	7.02E-03	6.54E-01
HLA.C	0.723	9.758	7.02E-03	6.54E-01
ARL4D	0.870	4.566	7.03E-03	6.54E-01
STAP1	1.021	-4.093	7.06E-03	6.54E-01
ACADVL	-0.510	8.720	7.08E-03	6.54E-01
SSX6	1.655	-4.307	7.16E-03	6.54E-01
RHCE	1.327	-1.002	7.17E-03	6.54E-01
IL13	1.132	-4.756	7.22E-03	6.54E-01
KLHL32	1.016	-1.451	7.28E-03	6.54E-01
ZNF467	-1.012	2.787	7.30E-03	6.54E-01
SLFN14	0.755	-4.856	7.33E-03	6.54E-01
FAM75A2	0.708	-5.498	7.36E-03	6.54E-01
TIMM8A	0.520	3.485	7.40E-03	6.54E-01
CD177	1.434	-3.277	7.41E-03	6.54E-01
MAP6D1	0.615	-0.127	7.43E-03	6.54E-01
CCDC28A	0.439	4.374	7.43E-03	6.54E-01
LOC100125556	0.666	0.930	7.45E-03	6.54E-01
UGT1A7	1.828	-3.480	7.48E-03	6.54E-01
TYRP1	1.807	-3.283	7.50E-03	6.54E-01
C1orf180	0.429	-5.628	7.50E-03	6.54E-01
SEPT14	1.416	-4.978	7.53E-03	6.54E-01
MAGEA9B	1.447	-4.789	7.53E-03	6.54E-01
C7orf13	-0.678	2.105	7.57E-03	6.55E-01
TRIM38	0.542	3.902	7.62E-03	6.56E-01
SSX4	2.289	-3.918	7.71E-03	6.62E-01
ANO2	1.427	-1.891	8.02E-03	6.81E-01
SH3PXD2A	-0.611	4.934	8.05E-03	6.81E-01
GRN	0.466	8.283	8.11E-03	6.81E-01
PI15	1.794	-0.941	8.13E-03	6.81E-01
IFIH1	0.643	4.118	8.14E-03	6.81E-01
FGF22	0.723	-5.198	8.20E-03	6.81E-01
ITGB7	0.867	2.388	8.21E-03	6.81E-01
NF2	-0.422	4.604	8.23E-03	6.81E-01
GABRA3	1.772	-4.042	8.25E-03	6.81E-01
C10orf32	-0.511	5.613	8.29E-03	6.81E-01
LYPD1	-1.819	2.070	8.30E-03	6.81E-01
IQCF1	0.690	-5.404	8.40E-03	6.83E-01
MAGEA1	2.945	-0.442	8.40E-03	6.83E-01
DIP2C	-0.758	4.339	8.44E-03	6.84E-01

C12orf74	0.936	-3.823	8.50E-03	6.86E-01
MLH3	-0.436	3.925	8.64E-03	6.90E-01
HOXA13	2.077	-0.132	8.65E-03	6.90E-01
CD79B	0.668	1.199	8.72E-03	6.90E-01
RXRG	1.791	-2.221	8.73E-03	6.90E-01
CSAG2	2.238	-3.630	8.75E-03	6.90E-01
PSMD12	0.384	5.038	8.78E-03	6.90E-01
NEDD9	-0.810	5.057	8.80E-03	6.90E-01
LRRC37A	-0.572	2.860	8.82E-03	6.90E-01
FITM1	1.006	0.041	8.89E-03	6.92E-01
PTCHD3	1.108	-4.976	8.94E-03	6.92E-01
FLJ36000	1.056	-5.035	8.94E-03	6.92E-01
PSMD3	0.324	6.856	9.04E-03	6.97E-01
LOXHD1	-1.005	-3.768	9.08E-03	6.97E-01
ZNF883	1.535	-1.643	9.28E-03	7.10E-01
SDCBP	0.523	7.064	9.32E-03	7.10E-01
FGFR2	-2.248	3.879	9.45E-03	7.17E-01
MAGEA10	1.122	-5.184	9.47E-03	7.17E-01
C15orf54	-0.660	-4.976	9.52E-03	7.18E-01
C3orf14	1.478	-0.521	9.59E-03	7.20E-01
HTATSF1	0.421	5.706	9.63E-03	7.21E-01
TRPV1	-0.533	3.358	9.68E-03	7.22E-01
DSC1	0.647	-5.107	9.94E-03	7.36E-01
PARK7	0.426	7.393	9.97E-03	7.36E-01
IL28A	0.268	-5.778	9.99E-03	7.36E-01

Table S2. Differentially expressed genes between 18 AA/Black and 14 White HCC samples collected in-house. "logFC" - log2 fold-change, "AveExpr" - log2 average expression, "P.Value" and "adj.P.Val" - raw and FDR-adjusted p-values.

Gene	logFC	AveExpr	P.Value	adj.P.Val
TMEM100	-3.366	2.951	4.86E-09	7.77E-05
IGF2	-5.123	7.878	8.69E-09	7.77E-05
RPS4XP22	-2.790	2.273	4.28E-08	2.55E-04
SLC16A12	-2.946	2.698	1.57E-07	7.03E-04
STEAP4	-2.570	5.430	5.39E-07	1.93E-03
MTND1P23	4.057	4.609	1.52E-06	3.71E-03
RPS28	-1.911	3.521	1.76E-06	3.71E-03
OLFM4	-5.894	6.990	1.84E-06	3.71E-03
CHN2-AS1	-1.254	1.248	1.89E-06	3.71E-03
PDE1C	-3.294	3.353	2.08E-06	3.71E-03
PCSK1	-2.950	3.768	2.73E-06	4.43E-03
LINC00176	-3.205	3.785	3.37E-06	5.02E-03
SEPT4	-2.257	5.505	5.15E-06	7.08E-03
MYH3	-2.694	2.665	5.64E-06	7.20E-03
FIGF	-2.833	2.189	6.27E-06	7.47E-03
KL	-1.315	1.056	1.28E-05	1.30E-02
OAS3	1.806	6.430	1.28E-05	1.30E-02
CORIN	-2.267	1.453	1.31E-05	1.30E-02
HS1BP3-IT1	-2.161	1.787	1.61E-05	1.51E-02
PPM1E	2.487	2.097	2.64E-05	2.28E-02
ARL4A	-1.619	4.094	2.67E-05	2.28E-02
PTPRN2	-1.941	2.247	2.87E-05	2.34E-02
UPP2	-3.114	3.206	3.18E-05	2.38E-02
IFI6	2.441	8.114	3.22E-05	2.38E-02
ASS1P1	-1.704	3.298	3.37E-05	2.38E-02
LTF	-2.317	1.331	3.46E-05	2.38E-02
SNAI2	-1.607	3.793	3.74E-05	2.41E-02
ASRGL1	1.794	2.622	3.77E-05	2.41E-02
PARM1	-1.918	3.683	4.82E-05	2.92E-02
OAS1	1.637	5.400	4.90E-05	2.92E-02
MX1	1.935	6.324	5.06E-05	2.92E-02
MT1X	-2.181	4.661	5.48E-05	3.06E-02
FITM2	-1.084	4.686	7.72E-05	4.04E-02
FCGR3B	-2.381	3.004	7.72E-05	4.04E-02
CYP1B1	-1.712	6.038	7.91E-05	4.04E-02
TRPC6	-1.093	1.304	9.23E-05	4.59E-02
MOCS2	-0.956	6.100	9.65E-05	4.66E-02
PDK4	-1.840	8.457	9.91E-05	4.66E-02

MYLK	-1.016	7.321	1.38E-04	6.13E-02
OAT	-2.439	7.525	1.39E-04	6.13E-02
SMOC1	-1.150	5.416	1.41E-04	6.13E-02
FKBP5	-1.354	7.807	1.50E-04	6.37E-02
RPL12P14	-1.621	-0.298	1.58E-04	6.41E-02
C2CD4D	1.138	1.200	1.61E-04	6.41E-02
ASS1P11	-1.821	3.803	1.61E-04	6.41E-02
SYBU-AS1	-2.006	1.269	1.77E-04	6.87E-02
CYP1B1-AS1	-0.993	0.538	1.82E-04	6.92E-02
PARP12	0.903	5.734	1.94E-04	7.05E-02
ASS1P7	-1.790	1.103	1.95E-04	7.05E-02
TIPARP-AS1	-1.518	1.442	2.05E-04	7.05E-02
ASS1P13	-1.676	3.730	2.12E-04	7.05E-02
C19orf71	-1.019	0.037	2.14E-04	7.05E-02
MT-ND3	-1.045	9.506	2.20E-04	7.05E-02
ZFAND5	-1.144	8.280	2.24E-04	7.05E-02
ASS1	-1.593	8.621	2.34E-04	7.05E-02
OAS2	1.801	6.101	2.35E-04	7.05E-02
IFI44L	2.255	6.110	2.38E-04	7.05E-02
ATP1A2	-1.564	1.032	2.41E-04	7.05E-02
OXNAD1	-0.720	5.040	2.42E-04	7.05E-02
ICA1	1.315	3.810	2.45E-04	7.05E-02
IFITM3P2	1.625	2.059	2.46E-04	7.05E-02
MMD	0.888	4.617	2.49E-04	7.05E-02
TRNP1	2.341	4.117	2.52E-04	7.05E-02
SLFN12	-1.189	2.882	2.52E-04	7.05E-02
ISG15	1.973	6.601	2.62E-04	7.21E-02
ASS1P14	-1.655	3.183	2.72E-04	7.36E-02
TREH	-2.021	1.578	2.86E-04	7.48E-02
ENSG00000279207	-0.970	3.420	2.89E-04	7.48E-02
PHYHD1	-1.584	2.316	2.91E-04	7.48E-02
ASS1P3	-1.652	2.587	2.95E-04	7.48E-02
CMPK2	1.914	4.533	3.05E-04	7.48E-02
EDA2R	-1.436	1.100	3.05E-04	7.48E-02
LGALS8-AS1	1.329	-0.290	3.05E-04	7.48E-02
USP18	1.148	2.289	3.18E-04	7.68E-02
CIB2	1.881	1.255	3.29E-04	7.83E-02
LDHB	-1.569	5.689	3.33E-04	7.83E-02
ENSG00000234639	-1.109	-0.605	3.41E-04	7.91E-02
IL20RB	-1.909	0.977	3.56E-04	8.16E-02
RASL10B	-2.833	5.120	3.75E-04	8.50E-02
COL12A1	-1.657	6.003	3.92E-04	8.76E-02
RPL15P18	1.856	0.587	4.07E-04	8.98E-02

RAB37	-1.235	3.873	4.25E-04	9.28E-02
TYMP	1.082	6.330	4.61E-04	9.87E-02
ASPSCR1	-1.536	6.175	4.64E-04	9.87E-02
CYP1A1	-2.573	5.778	4.72E-04	9.92E-02
SS18L1	-0.855	5.469	4.77E-04	9.92E-02
ZBED3	-0.931	4.248	4.83E-04	9.93E-02
SEPT3	2.272	1.190	4.95E-04	1.00E-01
MTMR7	1.868	2.972	5.00E-04	1.00E-01
ASS1P9	-1.576	2.139	5.15E-04	1.02E-01
PLA2G4C	0.995	4.030	5.22E-04	1.03E-01
NXN	-1.016	2.294	5.43E-04	1.05E-01
ATOH8	-1.585	3.237	5.78E-04	1.11E-01
IFI27	2.520	9.388	5.84E-04	1.11E-01
ADPRM	-0.909	3.041	6.18E-04	1.15E-01
DDIT3	1.177	4.032	6.22E-04	1.15E-01
FAM21EP	1.253	-0.602	6.23E-04	1.15E-01
SRGAP3	-1.015	3.346	6.35E-04	1.16E-01
STRA13	-1.657	6.359	6.41E-04	1.16E-01
MT1XP1	-1.874	2.041	6.65E-04	1.17E-01
SPATA18	-1.971	2.856	6.70E-04	1.17E-01
ENSG00000267904	-0.946	-0.236	6.72E-04	1.17E-01
FAS	-1.007	4.640	6.76E-04	1.17E-01
C2orf82	-2.003	4.187	6.87E-04	1.18E-01
SLC22A10	2.080	4.421	7.02E-04	1.19E-01
RAB11FIP1	1.055	5.056	7.03E-04	1.19E-01
C3orf58	-0.950	5.158	7.16E-04	1.20E-01
LGALS3BP	1.331	7.110	7.35E-04	1.21E-01
IFIT2	1.378	5.831	7.36E-04	1.21E-01
DKK3	-1.698	5.054	7.68E-04	1.25E-01
ZBTB8OSP2	1.158	0.405	7.78E-04	1.25E-01
ENSG00000258320	-1.761	0.823	7.82E-04	1.25E-01
SLC16A14	-2.395	4.785	7.97E-04	1.26E-01
BACH2	1.416	3.324	8.11E-04	1.27E-01
ADM2	1.382	2.212	8.40E-04	1.31E-01
NPR3	-1.429	4.325	8.64E-04	1.33E-01
GAS6-AS1	-1.458	2.013	8.84E-04	1.35E-01
MRPS21P1	1.053	-0.498	8.97E-04	1.36E-01
C1orf61	3.070	2.066	9.06E-04	1.36E-01
NKAPL	-1.574	-0.703	9.38E-04	1.40E-01
SERHL2	-1.582	0.721	9.51E-04	1.40E-01
LY6E	1.629	5.593	9.56E-04	1.40E-01
LOC100131496	-1.033	0.113	9.70E-04	1.40E-01
RORA	-1.028	7.661	9.71E-04	1.40E-01

SORD	-1.397	7.079	9.88E-04	1.41E-01
TGFB1I1	-0.838	2.315	1.02E-03	1.44E-01
SREBF1	1.016	6.092	1.03E-03	1.44E-01
GGH	-1.246	8.057	1.03E-03	1.44E-01
SLC22A4	-1.162	1.756	1.06E-03	1.45E-01
COL4A3	1.908	2.515	1.06E-03	1.45E-01
ARSK	-0.875	3.622	1.07E-03	1.45E-01
ANKRD37	-1.641	3.286	1.08E-03	1.45E-01
PDE8B	-0.907	2.315	1.08E-03	1.45E-01
ENSG00000253223	0.763	1.394	1.09E-03	1.45E-01
ATP10A	-1.809	2.668	1.15E-03	1.52E-01
ENSG00000234936	-1.240	-0.730	1.16E-03	1.52E-01
RPS2P44	0.883	-0.450	1.16E-03	1.52E-01
ADPRHL1	1.594	1.403	1.20E-03	1.55E-01
ENSG00000273893	-1.086	-0.308	1.21E-03	1.56E-01
IFIT3	1.339	5.778	1.22E-03	1.56E-01
ZNF165	1.402	2.339	1.24E-03	1.56E-01
GPR35	2.017	1.857	1.25E-03	1.56E-01
SPNS2	-1.002	2.740	1.25E-03	1.56E-01
CDH11	-2.020	4.801	1.26E-03	1.56E-01
ELL2P1	-0.938	3.195	1.27E-03	1.56E-01
CDCA4P4	1.294	0.714	1.28E-03	1.56E-01
NPY1R	-1.581	1.815	1.29E-03	1.57E-01
HCP5	0.982	5.302	1.30E-03	1.57E-01
SULT1C4	2.380	3.010	1.31E-03	1.57E-01
KCNE4	-1.653	3.001	1.35E-03	1.61E-01
CYP3A5	1.571	9.509	1.40E-03	1.66E-01
SLC16A4	-1.436	3.835	1.42E-03	1.67E-01
GDF15	1.369	4.743	1.48E-03	1.71E-01
MOCS2-DT	-1.052	2.096	1.48E-03	1.71E-01
ACAA1	-0.963	7.008	1.49E-03	1.71E-01
SETD9	-0.822	3.338	1.50E-03	1.72E-01
PGM5	-1.279	2.602	1.51E-03	1.73E-01
ALMS1-IT1	0.939	1.039	1.59E-03	1.80E-01
GPR153	-1.493	3.013	1.62E-03	1.82E-01
MTCO1P12	1.813	6.143	1.64E-03	1.82E-01
ELL2	-0.889	7.916	1.65E-03	1.82E-01
ENSG00000261118	-0.949	-0.229	1.66E-03	1.82E-01
ASS1P5	-1.505	-0.077	1.67E-03	1.82E-01
MMP24	-1.395	2.673	1.67E-03	1.82E-01
PSPN	-0.714	1.006	1.69E-03	1.83E-01
KANTR	1.449	2.052	1.72E-03	1.84E-01
CA2	-1.204	5.417	1.74E-03	1.84E-01

ASPA	-1.242	1.930	1.74E-03	1.84E-01
FARP2	0.484	5.219	1.74E-03	1.84E-01
ATXN7L1	-0.737	3.748	1.76E-03	1.86E-01
RNF43	-1.406	5.709	1.82E-03	1.89E-01
PLIN2	0.990	7.658	1.83E-03	1.89E-01
QPCT	1.826	2.547	1.83E-03	1.89E-01
IFIH1	0.849	5.554	1.88E-03	1.91E-01
GABARAPL1	-1.245	7.003	1.88E-03	1.91E-01
PDLIM1	-0.721	6.069	1.88E-03	1.91E-01
HERC6	1.273	4.681	1.90E-03	1.92E-01
TIGD7	0.915	1.667	1.93E-03	1.94E-01
ZNF804A	2.225	1.169	1.96E-03	1.95E-01
CTNNBL1	-0.936	5.673	1.97E-03	1.95E-01
GRHPR	-0.965	7.822	1.97E-03	1.95E-01
UBXN6	-0.809	6.444	2.03E-03	2.00E-01
PTBP2	0.759	4.930	2.04E-03	2.00E-01
CACNA2D3	-1.409	0.423	2.08E-03	2.02E-01
TRPV4	-1.865	2.199	2.10E-03	2.02E-01
EFHD1	-1.610	2.347	2.10E-03	2.02E-01
JAM2	-0.906	2.860	2.13E-03	2.03E-01
XAF1	1.273	4.779	2.13E-03	2.03E-01
AFAP1L2	-0.916	1.607	2.16E-03	2.04E-01
TMEM245	-0.940	8.295	2.22E-03	2.09E-01
KLHDC1	-0.798	2.459	2.27E-03	2.13E-01
ODF3B	0.788	4.026	2.30E-03	2.13E-01
DDX60L	0.900	5.740	2.32E-03	2.13E-01
NFIC	-1.032	6.841	2.32E-03	2.13E-01
NBPF3	0.871	2.311	2.34E-03	2.13E-01
OSBPL11	-0.674	5.725	2.36E-03	2.13E-01
ENSG00000267745	-1.075	-0.749	2.37E-03	2.13E-01
TPRG1	-1.421	4.528	2.37E-03	2.13E-01
CCT6B	-1.215	2.086	2.39E-03	2.13E-01
JAZF1	-0.798	4.634	2.39E-03	2.13E-01
SLC13A3	-2.018	5.890	2.39E-03	2.13E-01
ASS1P10	-1.403	1.845	2.41E-03	2.13E-01
HOOK1	0.843	6.460	2.43E-03	2.13E-01
SPG20	-1.043	4.165	2.44E-03	2.13E-01
ICAM1	1.037	5.747	2.45E-03	2.13E-01
LRRC17	-1.034	1.582	2.46E-03	2.13E-01
IFITM1	1.421	4.086	2.49E-03	2.15E-01
HCG4P7	0.903	0.178	2.52E-03	2.17E-01
IFIT1	1.734	6.916	2.55E-03	2.18E-01
TFPI2	1.811	1.964	2.58E-03	2.19E-01

OCLN	0.866	5.760	2.62E-03	2.22E-01
ASS1P12	-1.069	0.762	2.63E-03	2.22E-01
CYGB	-1.024	2.781	2.66E-03	2.23E-01
GREB1	-1.595	4.174	2.67E-03	2.23E-01
ST3GAL6	-0.987	5.499	2.73E-03	2.27E-01
ENSG00000271500	0.739	1.771	2.75E-03	2.27E-01
MTCO2P12	1.847	3.155	2.76E-03	2.27E-01
HLA-F	0.883	6.754	2.78E-03	2.27E-01
UGT1A2P	-1.864	3.127	2.78E-03	2.27E-01
AASS	-1.333	6.730	2.82E-03	2.29E-01
RPS20P35	1.143	2.655	2.86E-03	2.31E-01
EDNRB	-0.748	5.144	2.87E-03	2.31E-01
HERC2P3	1.412	1.674	2.92E-03	2.34E-01
LINC00997	-0.744	0.484	2.93E-03	2.34E-01
AHCYP4	-0.883	-0.358	2.95E-03	2.34E-01
CYB5D2	-0.909	4.122	2.96E-03	2.34E-01
CAPS	-1.112	2.986	2.99E-03	2.35E-01
UHRF1BP1	0.591	5.893	3.04E-03	2.38E-01
HLA-H	1.020	5.892	3.05E-03	2.38E-01
EEF2	-0.619	10.480	3.07E-03	2.38E-01
RAG1	-1.786	1.883	3.09E-03	2.38E-01
TMEM44-AS1	-0.991	2.453	3.10E-03	2.38E-01
FAM174B	-0.815	2.408	3.11E-03	2.38E-01
FAM169A	-1.463	5.581	3.15E-03	2.41E-01
TSPAN11	-1.196	0.709	3.21E-03	2.44E-01
IFI44	1.445	5.708	3.26E-03	2.46E-01
SLC45A1	-1.237	-0.026	3.26E-03	2.46E-01
SSBP3P1	-1.059	2.821	3.34E-03	2.50E-01
ENSG00000278383	0.837	0.651	3.36E-03	2.50E-01
GPD2	0.714	5.476	3.36E-03	2.50E-01
GPX7	-1.339	2.593	3.38E-03	2.51E-01
OSER1-AS1	-0.926	3.189	3.41E-03	2.52E-01
FRZB	-1.013	2.692	3.45E-03	2.53E-01
CD248	-1.098	2.089	3.46E-03	2.53E-01
SBSPON	-1.330	1.505	3.47E-03	2.53E-01
JUN	0.889	6.945	3.49E-03	2.53E-01
KCNT2	1.249	5.524	3.57E-03	2.58E-01
RP11-644F5.10	-0.888	-0.390	3.57E-03	2.58E-01
STRIP2	1.498	3.057	3.60E-03	2.58E-01
LEKR1	-0.892	1.110	3.61E-03	2.58E-01
FNTA	0.608	5.402	3.64E-03	2.60E-01
SLITRK4	-1.811	0.981	3.73E-03	2.63E-01
MUC6	-2.712	3.916	3.73E-03	2.63E-01

GABPB1-AS1	-0.635	4.828	3.74E-03	2.63E-01
PLA2G4A	-1.967	3.411	3.76E-03	2.63E-01
ESR1	-1.316	4.478	3.80E-03	2.65E-01
MCOLN3	2.392	2.224	3.80E-03	2.65E-01
RPL21P65	0.876	-0.802	3.86E-03	2.67E-01
EFNA3	1.340	0.878	3.89E-03	2.69E-01
GALNT14	1.446	-0.103	3.93E-03	2.70E-01
HOGA1	-1.559	3.401	3.94E-03	2.70E-01
DNAAF1	-1.606	-0.135	3.97E-03	2.71E-01
TSPAN5	-1.295	3.402	4.00E-03	2.72E-01
VNN1	1.942	6.878	4.02E-03	2.73E-01
ARHGAP44	-1.106	1.235	4.06E-03	2.74E-01
LUM	-2.026	6.978	4.14E-03	2.77E-01
MT1E	-2.043	5.667	4.14E-03	2.77E-01
YPEL3-DT	1.116	0.103	4.15E-03	2.77E-01
ECHDC3	-0.991	5.929	4.35E-03	2.87E-01
PDLIM1P1	-0.727	-0.166	4.35E-03	2.87E-01
IL1R2	-0.986	3.236	4.36E-03	2.87E-01
ENSG00000275880	-1.502	0.943	4.37E-03	2.87E-01
CCNE1	1.793	2.954	4.40E-03	2.88E-01
HSD17B8	-1.263	5.542	4.43E-03	2.88E-01
SP140L	0.592	4.082	4.45E-03	2.88E-01
RPS4XP7	-1.197	0.050	4.45E-03	2.88E-01
COL10A1	-1.376	4.084	4.48E-03	2.88E-01
GBAP1	0.729	0.689	4.49E-03	2.88E-01
PDXK	-0.612	6.413	4.54E-03	2.91E-01
PDXK	-0.612	6.413	4.54E-03	2.91E-01
BST2	1.030	7.224	4.61E-03	2.94E-01
FCRL6	-1.032	0.549	4.64E-03	2.95E-01
SESN1	-0.686	5.175	4.67E-03	2.96E-01
